# Improving selection decisions with mating information by accounting for Mendelian sampling variances looking two generations ahead

**DOI:** 10.1186/s12711-024-00899-2

**Published:** 2024-05-21

**Authors:** Tobias A. M. Niehoff, Jan ten Napel, Piter Bijma, Torsten Pook, Yvonne C. J. Wientjes, Bernadett Hegedűs, Mario P. L. Calus

**Affiliations:** Animal Breeding and Genomics, Wageningen University and Research, Droevendaalsesteeg 1, 6700AH Wageningen, The Netherlands

## Abstract

**Background:**

Breeding programs are judged by the genetic level of animals that are used to disseminate genetic progress. These animals are typically the best ones of the population. To maximise the genetic level of very good animals in the next generation, parents that are more likely to produce top performing offspring need to be selected. The ability of individuals to produce high-performing progeny differs because of differences in their breeding values and gametic variances. Differences in gametic variances among individuals are caused by differences in heterozygosity and linkage. The use of the gametic Mendelian sampling variance has been proposed before, for use in the usefulness criterion or Index5, and in this work, we extend existing approaches by not only considering the gametic Mendelian sampling variance of individuals, but also of their potential offspring. Thus, the criteria developed in this study plan one additional generation ahead. For simplicity, we assumed that the true quantitative trait loci (QTL) effects, genetic map and the haplotypes of all animals are known.

**Results:**

In this study, we propose a new selection criterion, ExpBVSelGrOff, which describes the genetic level of selected grand-offspring that are produced by selected offspring of a particular mating. We compare our criterion with other published criteria in a stochastic simulation of an ongoing breeding program for 21 generations for proof of concept. ExpBVSelGrOff performed better than all other tested criteria, like the usefulness criterion or Index5 which have been proposed in the literature, without compromising short-term gains. After only five generations, when selection is strong (1%), selection based on ExpBVSelGrOff achieved 5.8% more commercial genetic gain and retained 25% more genetic variance without compromising inbreeding rate compared to selection based only on breeding values.

**Conclusions:**

Our proposed selection criterion offers a new tool to accelerate genetic progress for contemporary genomic breeding programs. It retains more genetic variance than previously published criteria that plan less far ahead. Considering future gametic Mendelian sampling variances in the selection process also seems promising for maintaining more genetic variance.

**Supplementary Information:**

The online version contains supplementary material available at 10.1186/s12711-024-00899-2.

## Background

Breeding programs generate genetic gain by selective breeding, which traditionally relies on selecting the best animals as parents of the next generation. Since the advent of genomic prediction, the most widely used criterion to rank and select animals is the genomic estimated breeding value (GEBV). In general, selection based on estimated breeding values maximizes the average genetic level in the next generation.

Usually, the implicit primary aim of contemporary commercial breeding programs is to maximize the genetic level of animals from which genetic material, such as semen, is sold. This is different to maximizing the population average breeding value. Thus, an implicit goal of breeding programs is to identify individuals to be used as parents of the next generation that generate more very good offspring. This is different from selecting the best animals as parents since the parents do not necessarily need to have the genetics for high performances themselves, but they should have the ability to produce some offspring genotypes that can be expected to show very high performance. The top performing offspring may then be used for production or for dissemination of genetic material to production farms as in the case of hybrid breeding. In other words, breeding programs have two goals: (A) selecting top individuals to disseminate genetic progress to production levels and (B) selection of individuals as parents to breed the next generation of the nucleus, i.e., improve the breeding population. The best group of animals to select to serve one goal, is not necessarily also the best group of animals to serve the other goal. This concept to distinguish between selection of animals to serve the market and selection of animals to improve the population is highly related to the two-part breeding strategy that Gaynor et al. [1] proposed for the development of inbred lines in plant breeding.

Several indices for the selection of individuals as parents have been proposed in animal breeding research [2–4]. All of these proposed criteria combine the breeding value with the gametic Mendelian sampling variance (gametic MSV) of an animal. This is because maximizing these two parameters maximizes the probability of generating a top performing offspring from the respective selection candidate. The gametic Mendelian sampling variance is the variance of breeding values among the gametes produced by an animal. Musa and Reinsch [5] showed in a simulation study that selection based on Index5, combining the breeding value and gametic MSV as developed by Bijma et al. [3], achieved more genetic gain and preserved more genetic variation compared to truncation selection solely based on breeding values.

Under the infinitesimal model, the theoretical expectation of the gametic MSV is $gamMSV=0.25 \left(1-F\right){\sigma }_{A}^{2}$, where $F$ is the inbreeding coefficient that is estimated relative to a base population, and ${\sigma }_{A}^{2}$ is the additive genetic variance in this unselected randomly mating base population [6]. This expectation is based on the assumption that variation in gametic MSV is only due to variation in inbreeding coefficients, and seems to be a poor predictor of the actual variation observed in offspring as found in cattle [7, 8]. This is in part because the gametic MSV is not only influenced by the homozygosity of an animal but also by the linkage phase, the distribution of quantitative trait loci (QTL) across the genome, and the QTL effect sizes. In the genomics era, haplotypes can be reconstructed with phasing software and estimated SNP effects from genomic prediction models can be used to approximate QTL effects. Paired with linkage maps to calculate recombination frequencies between loci, gametic MSV can be computed algebraically [2, 5, 9–14] or approximated by simulating gametes [7, 15].

In plant breeding, the inclusion of offspring variances for selection decisions has been suggested by Schnell and Utz [16]. Schnell and Utz [16] proposed the usefulness criterion for variety development, which describes the expected breeding value of the best performing line of a cross. The usefulness criterion has received some attention by the plant breeding community [9, 11, 13, 17–21]. To our knowledge, all previously published selection criteria that use Mendelian sampling variances focus on maximizing genetic gain of the top individuals in the next generation and have been shown to outperform truncation selection based on breeding values [3, 5, 11, 22], be it in an animal breeding context or a plant breeding context.

In this study, we developed new criteria that not only consider the offspring generation but also the grand-offspring generation, i.e., our criteria look one more generation ahead to accelerate genetic gain. With respect to the goals of breeding programs mentioned above, we see yet another goal, namely the selection of grandparents which produce good parents to then produce top performing offspring to serve the market. We compare our criteria with other already proposed criteria [3, 7, 16]. Since accelerating genetic response tends to have the disadvantage of compromising genetic diversity, we analyze several relevant diversity parameters to verify the impact of our proposed criteria.

## Methods

This section starts with an illustration of why looking two generations ahead may be beneficial for selecting parents, followed by the description of the selection criteria considered in this study. We then explain how the gametic MSV of offspring individuals can be calculated. We end with the description of the simulation setup of the breeding program in which we tested the proposed selection criteria.

### Motivation to look two generations ahead

In the following, we will make use of the abbreviation F1s and F2s which are commonly used in plant breeding with F1s being the direct offspring of parents, i.e., separated by one meiosis, and F2s being the offspring of F1s, i.e., separated by two meiosis from the parents.

**Fig. 1:**
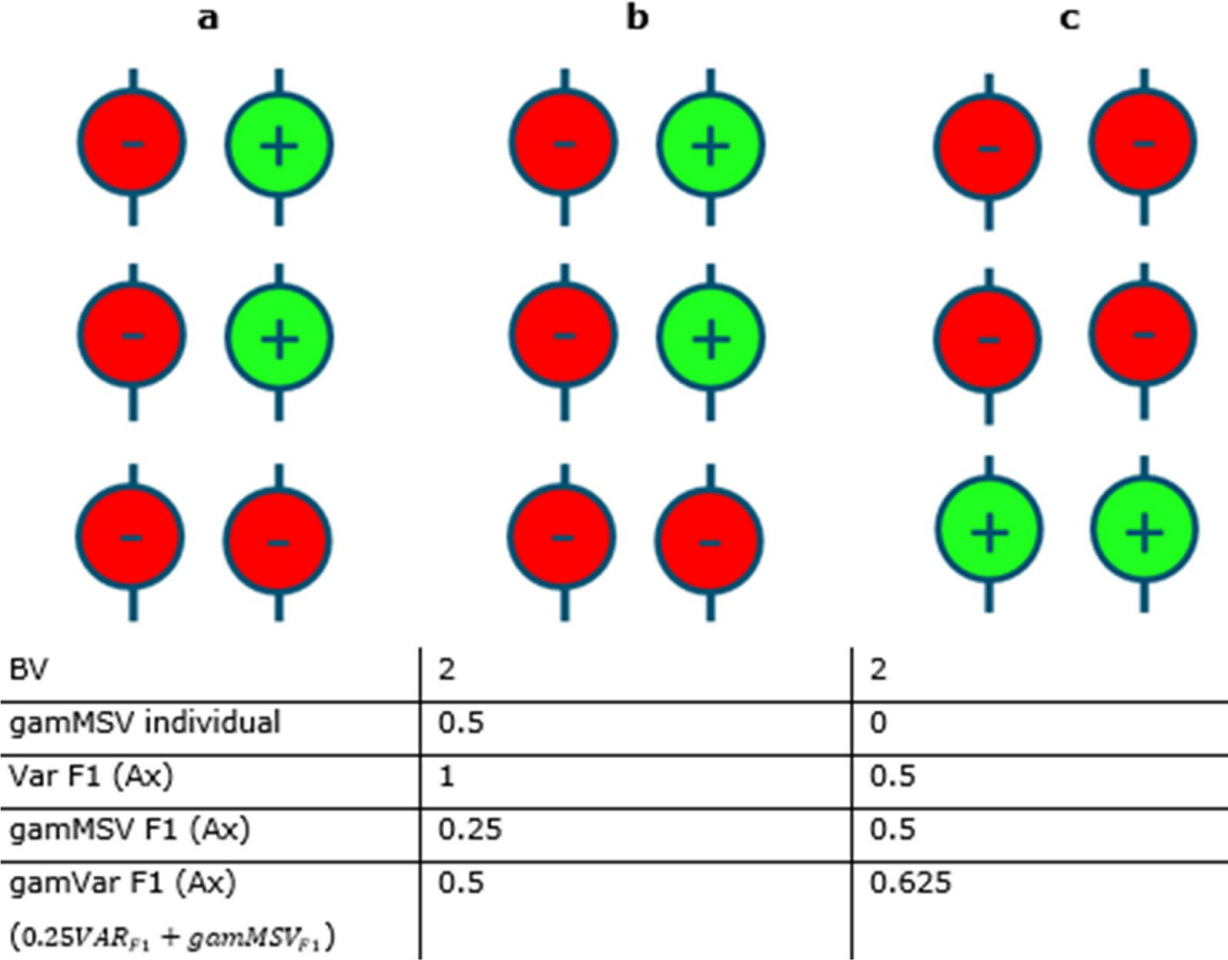
Illustrative example for the mating choice problem. Green circles indicate beneficial variants with an effect of + 1 and red circles indicate the unfavorable variants with effect size 0. Shown are three independent loci with two alleles each. “Var F1 (Ax)” refers to the variance of breeding values of the F1 of the mating of the respective individual to individual A. “gamVar F1” refers to the variance of breeding values among gametes produced by the F1 individuals. Note that the variance of breeding values of the F1 is influencing this variance together with the average gametic MSV of an F1 individual

Consider the following illustrative example (Fig. 1): let us imagine that individuals B and C can be chosen to be mated to individual A. All three individuals have the same breeding value, and therefore the expected performance of random offspring is the same for the mating AxB and AxC. Thus, the possible matings cannot be distinguished based on their parent average breeding value. However, if the goal is to produce an outstanding individual with a very high breeding value in the next generation, mating AxB should be preferred because the offspring of mating AxB show a larger variance in their breeding values than offspring of mating AxC. This is because individual B has a larger gametic variance (0.5) than individual C (0) since individual C is fully homozygous. In other words, it is more likely to obtain an F1 individual with a high breeding value from the AxB mating than from the AxC mating.

However, if the goal is to produce the highest ranking gamete produced by the F1, which is equivalent to producing the highest ranking F2 individual, then mating AxC should be preferred. In other words, the probability to produce a gamete with a high breeding value is higher for offspring resulting from mating AxC than from mating AxB, which increases the probability to produce grand-offspring with very high breeding values. This is because the gametic variance of the F1 offspring is larger for mating AxC than for mating AxB.

The variance of all gametes produced by F1 offspring is larger for mating AxC (0.625) than for mating AxB (0.5). This simple example shows that the generation for which one wants to maximize gain, matters when choosing the parents. This example also shows that by considering two future generations, the optimum selection decision depends not only on properties of the individuals themselves but also on combinations of individuals, i.e., matings.

From a practical breeding perspective, selection will be applied in every generation. Strictly speaking, the variance of gametes produced by only the selected F1 offspring is of relevance when optimizing selection decisions for maximum gain in the second generation. For simplicity, we have used the variance of gametes produced by all F1 offspring in this illustrative example. How selection may be accounted for will be explained in the next section.

### Review of transmission of variance

This section reviews the quantitative genetic model for the prediction of variances of future generations that we use in this study. These variances are needed for our developed selection criteria. The interested reader is referred to Falconer and Mackay [23] pp. 201–204 for further explanation. For the variance of gametes produced by selected F1 individuals of a mating, not only the gametic MSV of F1 individuals has to be considered but also the variance among breeding values of all selected F1 individuals. The sum of the variance of gametes produced by sires and dams is the variance of their direct offspring (F1s). Under random mating, the variance of breeding values of the next generation can be modeled with Eq. (1):

1 $${\sigma }_{{BV}_{t+1}}^{2}=\frac{\left(1-{k}_{sires}\right){\sigma }_{{BV}_{{sires}_{t}}}^{2}}{4}+\frac{\left(1-{k}_{dams}\right){\sigma }_{{BV}_{{dams}_{t}}}^{2}}{4} +{\sigma }_{{gamMS}_{{sires}_{t}}}^{2}+{\sigma }_{{gamMS}_{{dams}_{t}}}^{2},$$

with $k=i*(i-x$), where ${\sigma }_{{gamMS}_{{sires}_{t}}}^{2}$ and ${\sigma }_{{gamMS}_{{dams}_{t}}}^{2}$ are the gametic Mendelian sampling variances of the sires and dams in the current generation ($t$) and ${\sigma }_{{BV}_{t+1}}^{2}$ is the variance of breeding values in the next generation ($t+1$). The term $k$ is the variance reduction coefficient and can be calculated based on the selection intensity $i$ and the normalized truncation selection point $x$. The term $(1-{k}_{sires}){\sigma }_{{BV}_{{sires}_{t}}}^{2}$ is the variance of breeding values of sires selected for the next generation, and $\left(1-{k}_{dams}\right){\sigma }_{{BV}_{{dams}_{t}}}^{2}$ is the corresponding term for dams. The term $\frac{\left(1-{k}_{sires}\right){\sigma }_{{BV}_{{sires}_{t}}}^{2}}{4}+\frac{\left(1-{k}_{dams}\right){\sigma }_{{BV}_{{dams}_{t}}}^{2}}{4}$ represents the variance of parent average breeding values.

For a specific mating of two individuals, the variance among their offspring only depends on the sum of the gametic MSV terms of the parents as both ${\sigma }_{{BV}_{{sires}_{t}}}^{2}$ and ${\sigma }_{{BV}_{{dams}_{t}}}^{2}$ will be zero due to the fixed choice of one sire and one dam. However, if the group of selected F1 offspring of the mating are to be mated again, for example to each other, then these terms in the F1 are not zero and need to be considered to calculate the variance of the next generation. We use the concept reviewed in this section to derive expected variances of grand-offspring.

### Overview of the selection criteria evaluated in this study

This section aims at conveying intuition about what the selection criteria used in this study describe before providing their formal definitions. We tested criteria that have been discussed in the literature as well as two new criteria. The key features, such as the focal generation for which the breeding values are maximized, to what group of individuals the absolute truncation selection point is specific, and the unit of selection, are summarized in Table 1.

**Table 1:** Overview of the selection criteria compared in this study

Focal generation	Criterion	Absolute threshold specific to	Unit of selection	References
Current	BV	Population	Individual	
Progeny	ExpBVSelOff	Family	Mating	Schnell and Utz [16], called “Usefulness criterion”
Progeny	ProbSelOff	Population	Mating	Segelke et al. [7]
Progeny	Index5	Family	Individual	Bijma et al. [3]
Grandprogeny	ExpBVSelGrOff	Family	Mating	This study
Grandprogeny	ProbSelGrPrOff	Population	Mating	This study

BV = breeding value, ExpBVSelOff = expected breeding value of selected offspring, ProbSelOff = probability to select offspring, ExpBVSelGrOff = expected breeding value of selected grandprogeny, ProbSelGrOff = probability to select grandprogeny. Index5 is the fifth index tested by Bijma et al. [3] and describes the linearized probability that offspring are selected

Selection based on breeding values aims at selecting a group of individuals whose average breeding value is the highest, which maximizes the average breeding value of the next generation.

For breeding of selfing crop species, Schnell and Utz [16] proposed to select crosses based on the “usefulness criterion”. In this study, we refer to it with ExpBVSelOff as an abbreviation for the “expected breeding value of selected offspring” of a particular mating as a more meaningful name. Since two parents are involved in a mating, ExpBVSelOff is a value that is expressed for a pair of individuals. We assumed the same percentage of selected offspring for every family to calculate ExpBVSelOff which results in family specific absolute truncation selection points.

**Fig. 2:**
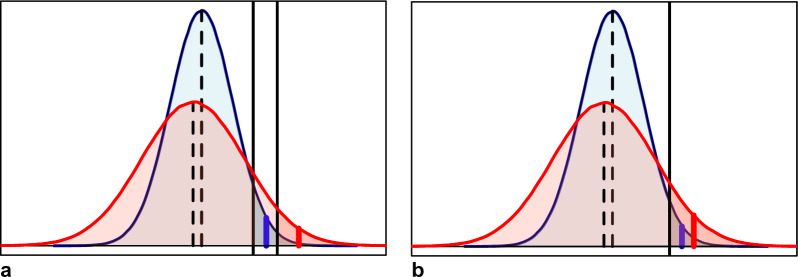
Hypothetical distribution of breeding values of F1 progeny of two matings (red and blue). The fractions of offspring with breeding values higher than the selection threshold (solid black line) are indicated in darker colors. The colored lines indicate the average breeding value of the selected fraction. **a** Using the same selection intensity for both matings as assumed in the calculation of ExpBVSelOff. **b** Using the same absolute truncation selection threshold for both matings as assumed in the calculation of ProbSelOff

Basing selection decisions on the probability of a mating to produce offspring that show a higher breeding value than the truncation selection point has been proposed in animal breeding by Segelke et al. [7]. We refer to this probability with ProbSelOff as a meaningful abbreviation for “probability for selected offspring”. Just as ExpBVSelOff, ProbSelOff is also expressed for a particular mating. The key difference with ExpBVSelOff is that for the calculation, the absolute truncation selection point is assumed to be the same for all matings. The difference between the two is shown in Fig. 2.

A few studies present selection criteria that are expressed for an individual but aim at maximizing the breeding value of top offspring of the individual without knowing its mate [2–4]. In this study, we used Index5 as suggested by Bijma et al. [3]. Index5 is a linearization of the probability of an individual to produce top offspring. Thus, conceptually, it describes the same information as ProbSelOff, but just for an individual instead of a mating. Expressing the quality on an individual basis is possible because the probability that offspring of an individual will be selected is the higher the individual’s breeding value and the higher the gametic MSV, irrespective of the mating partner. For a more thorough explanation, see Bijma et al. [3].

**Fig. 3:**
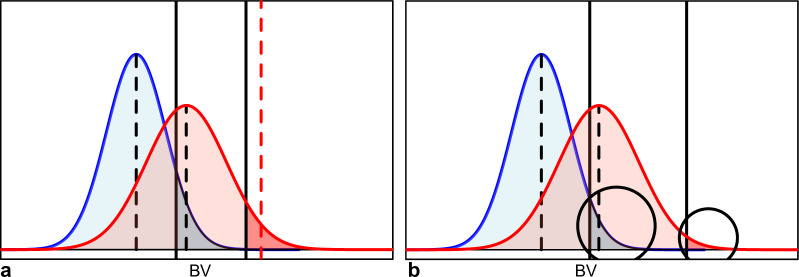
Visualization of the properties considered by the criteria looking two generations ahead. The blue distribution is the distribution of the breeding values of the offspring from one particular mating. The red distribution is the distribution of the breeding values of the grand-offspring of a mating which are produced by the selected offspring. Black dashed lines indicate the average breeding value in a group of individuals. Solid black lines indicate truncation selection points that are to be applied. **a** For ExpBVSelGrOff, the expected breeding value of the selected grand-offspring is indicated by a red dashed line. **b** For ProbSelGrOff, the probability that the offspring of a mating are selected is multiplied by the probability that the grand-offspring will be selected, which is conditional on knowing the selected offspring. Both probabilities are highlighted with black circles

Our new criteria do not aim at producing top offspring in the immediate next generation but in the second generation after the parental generation, i.e., the grand-offspring generation. The first criterion ExpBVSelGrOff represents the “expected breeding value of selected grand-offspring” (see plot a in Fig. 3). For ExpBVSelGrOff, we assume that selected offspring of a mating are mated at random to other selected individuals of that generation with unknown parentage. For ExpBVSelGrOff, we also assume that the selection intensity is the same for all fullsib offspring groups and grand-offspring groups, thus making the absolute selection threshold family specific (see Discussion for the motivation).

The other newly developed criterion is ProbSelGrOff. ProbSelGrOff describes the probability that grand-offspring of a mating will be selected. To calculate ProbSelGrOff, we multiply the probability that offspring are selected, which is identical to ProbSelOff, with the probability that grand-offspring produced by the selected offspring are selected. Both probabilities are indicated by large black circles in plot b of Fig. 3. The multiplication of both gives ProbSelGrOff. The selection thresholds used for ProbSelGrOff are the absolute truncation selection points that are to be applied to the entire population. Thus, the absolute truncation selection points to be applied in families are not family specific.

### Formal definition of selection criteria

In this section, we present the equations to calculate the six tested criteria.

Table 2 shows the explanations of the variables that are used throughout this section. We calculated the gametic Mendelian sampling variances ${\sigma }_{{gamMS}_{sire}}^{2}$, ${\sigma }_{{gamMS}_{dam}}^{2}$ and ${\sigma }_{{gamMS}_{animal}}^{2}$ with the analytical approach presented in Equation 5 in Musa and Reinsch [5]. The variances were calculated per chromosome and then summed across chromosomes, for computational efficiency.

**Table 2:** Definition of parameters

${BV}_{sire}$	Breeding value of a sire
${BV}_{dam}$	Breeding value of a dam
${BV}_{animal}$	Breeding value of an animal
${\sigma }_{{gamMS}_{sire}}^{2}$	Gametic Mendelian sampling variance of a sire
${\sigma }_{{gamMS}_{dam}}^{2}$	Gametic Mendelian sampling variance of a dam
${\sigma }_{{gamMS}_{animal}}^{2}$	Gametic Mendelian sampling variance of an animal
${\sigma }_{BV}^{2}$	Variance of breeding values in the population in the current generation
$\overline{BV }$	Average breeding value of the population in the current generation
${i}_{p}$	Selection intensity belonging to selected proportion $p$ of the population
${x}_{p}$	Normalized truncation selection point belonging to selected proportion $p$ of the population
${{\tau }_{p}}_{O}$	Absolute truncation selection point in the offspring generation based on selected proportion $p$ of the population; is equal to $\overline{BV }+\left({i}_{p}+{x}_{p}\right)\sqrt{{\sigma }_{BV}^{2}}$
${{\tau }_{p}}_{GO}$	Absolute truncation selection point in the grand-offspring generation based on selected proportion $p$ of the population; is equal to $\overline{BV }+\left({2i}_{p}+{x}_{p}\right)\sqrt{{\sigma }_{BV}^{2}}$
${\overline{BV} }_{sel \, off}$	Average breeding value of selected offspring of a mating. This is identical to ExpBVSelOff
${\overline{BV} }_{{sel \, ind}_{t}}$	Average breeding value of selected individuals of the population in the current generation ($t$); is equal to $\overline{BV }+{i}_{p}\sqrt{{\sigma }_{BV}^{2}}$
${\overline{BV} }_{{sel \, ind}_{t+1}}$	Average breeding value of selected individuals of the population in the next generation ($t+1$); is equal to $\overline{BV }+{2i}_{p}\sqrt{{\sigma }_{BV}^{2}}$
${\sigma }_{{gam}_{sel \, off}}^{2}$	Variance of breeding values of gametes produced by the group of selected offspring of a specific mating
${\overline{\sigma }}_{{gamMS}_{off}}^{2}$	Average gametic Mendelian sampling variance of offspring of a mating
${\sigma }_{FS}^{2}$	Variance of breeding values of full sib offspring of a mating; is equal to ${\sigma }_{{gamMS}_{sire}}^{2}+{\sigma }_{{gamMS}_{dam}}^{2}$
${\sigma }_{{gam}_{sel \, ind t}}^{2}$	Variance of breeding values of gametes produced by the group of selected individuals of the population in the current generation ($t$)
${\sigma }_{{gam}_{sel \, ind t+1}}^{2}$	Variance of breeding values of gametes produced by the group of selected individuals of the population in the next generation ($t+1$). Here, we assume ${\sigma }_{{gam}_{sel \, ind t+1}}^{2}={\sigma }_{{gam}_{sel \, ind t}}^{2}$
${\sigma }_{GO}^{2}$	Variance of breeding values of grand-offspring produced by selected offspring of a mating when mated to a random partner of selected animals of the population from the same generation; it is equal to ${\sigma }_{{gam}_{sel \, off}}^{2}+{\sigma }_{{gam}_{sel \, ind}}^{2}$

Note that ${\sigma }_{{gam}_{sel \, off}}^{2}$ is not the gametic MSV of a selected offspring individual, but the variance of breeding values of gametes produced by all selected offspring of a particular mating (Eq. (2)). The numerator of the first term of Eq. (2) represents the variance among selected offspring of a particular mating according to Bulmer [24] with $k$ as the variance reduction coefficient, $i$ as the selection intensity and $x$ as the normalized truncation selection point. For criterion ProbSelGrOff, note that $i$ and $x$ and thus $k$ differ per mating as the absolute truncation selection point is the same for all matings. Appendix 2 shows how $i$ and $x$ are derived for the calculation of criterion ProbSelGrOff. For ExpBVSelOff, $i$ and $x$ are identical to ${i}_{p}$ and ${x}_{p}$. Thus, $k$ is identical for all matings when calculating ExpBVSelOff.

2 $${\sigma }_{{gam}_{sel \, off}}^{2}= \frac{\left(1-k\right)*{\sigma }_{FS}^{2}}{4}+{\overline{\sigma }}_{{gamMS}_{off}}^{2},$$

with $$k=i*(i-x$$).

To our knowledge, no approach for the calculation of ${\overline{\sigma }}_{{gamMS}_{off}}^{2}$, i.e., the expected gametic MSV of offspring, has been published before. Thus, we propose a new approach for this, which is described in detail in the section “Calculation of gametic Mendelian sampling variance of offspring”.

The term ${\sigma }_{{gam}_{sel \, ind t+1}}^{2}$ is comparable to ${\sigma }_{{gam}_{sel \, off}}^{2}$ and describes the variance of gametes of all selected animals of the population in the offspring generation. Since calculating this property precisely is difficult, we assumed that the variance of breeding values of gametes produced by selected individuals in the next generation ($t+1$) is equal to the variance of breeding values of gametes in the current generation ($t$), so ${\sigma }_{{gam}_{sel \, ind t+1}}^{2}={\sigma }_{{gam}_{sel \, ind t}}^{2}$. In other words, we assumed that the average gametic MSV of undefined individuals selected from the population does not change in subsequent generations. The term ${\sigma }_{{gam}_{sel \, ind t}}^{2}$ can be calculated with Eq. (2) by substituting the relevant terms: instead of ${\sigma }_{FS}^{2}$ which describes the variance among fullsib offspring of a particular mating, we used the variance of breeding values among all selection candidates; and instead of ${\overline{\sigma }}_{{gamMS}_{off}}^{2}$, we used the average gametic MSV observed among all selection candidates as an approximation of the average gametic MSV of the animals in the population.

### Existing criteria

#### Breeding value (BV)

As a baseline selection criterion, we considered truncation selection on the breeding values.

#### Expected breeding value of selected offspring (ExpBVSelOff)

ExpBVSelOff is the expected breeding value of the selected fraction of progeny of a mating. We calculated the ExpBVSelOff criterion with Eq. (3):

3 $$ExpBVSelOff= \left(\frac{{BV}_{sire}+{BV}_{dam}}{2}\right)+{i}_{p}* \sqrt{{\sigma }_{FS}^{2}.}$$

#### Probability to select offspring (ProbSelOff)

The probability of a mating is defined as the fraction of progeny that is expected to have a higher breeding value than the absolute truncation selection threshold that is applied to the whole population. We calculated the probability with Eq. (4):

4 $$ProbSelOff=1-\Phi \left(\frac{{{\tau }_{p}}_{O}-\left(\frac{{BV}_{sire}+{BV}_{dam}}{2}\right)}{\sqrt{{\sigma }_{FS}^{2}}}\right),$$

where $\Phi$ is the cumulative normal distribution function. The term $\left(\frac{{BV}_{sire}+{BV}_{dam}}{2}\right)$ is the average breeding value of fullsib offspring. The term ${\sigma }_{FS}^{2}$ is the variance of breeding values among the fullsib offspring of the mating, and equals the sum of the gametic Mendelian sampling variances of the sire and dam.

#### Index5

This index was developed by Bijma et al. [3] and is the linearized probability of an individual to produce top-ranking offspring. It is the linearization of ProbSelOff, assuming an average mate. The calculation of the index value is shown in Eq. (5). Note that Index5 is a selection criterion that is expressed for an individual and does not require information about the mate of the individual, in contrast to ProbSelOff and ExpBVSelOff.

5 $${I}_{5}={BV}_{animal}+ {x}_{p}* \sqrt{{2*\sigma }_{{gamMS}_{animal}}^{2}}.$$

## Newly developed criteria

Our two newly developed criteria have in common that, in addition to breeding values, they not only consider the variance of breeding values of offspring of a mating but also the gametic MSV of these offspring. The motivation for this is that it is possible for a mating to produce very good offspring but these offspring themselves can have a low gametic MSV in which case they are unlikely to produce good grand-offspring, as in the case of a fullsib mating.

### Probability to select grand-offspring (ProbSelGrOff)

ProbSelGrOff expresses the probability for the grand-offspring of a mating to be selected, or in other words, to show a breeding value higher than the selection threshold applied to the grand-offspring generation. For ProbSelGrOff, the probability that the offspring of a mating will be selected, which is ProbSelOff (Eq. (4)), is multiplied with the probability that the selected offspring will themselves produce progeny that will be selected ($P\left({P}_{GO}|selected \, offspring\right)$). This second probability is conditional on knowing the average breeding value, variance of breeding values and gametic MSV of selected offspring. Equation (6) shows the calculations and Fig. 3 illustrates which probabilities are multiplied.

6 $$ProbSelGrOff=ProbSelOff*P\left({P}_{GO}|selected \, offspring\right).$$

For the calculation of ProbSelGrOff, we assume that the selected offspring of a particular mating are randomly mated to other individuals selected from the population in the same generation. Thus, the second term in Eq. (6) can be calculated with Eq. (7):

7 $$P\left({P}_{GO}|selected \, offspring\right)=1-\Phi \left(\frac{{{\tau }_{p}}_{GO}-\left(\frac{{\overline{BV} }_{sel \, off}+{\overline{BV} }_{{sel \, ind}_{t+1}}}{2}\right)}{\sqrt{{\sigma }_{{gam}_{sel \, off}}^{2}+{\sigma }_{{gam}_{sel \, ind t+1}}^{2}}}\right)$$

### Expected breeding value of selected grand-offspring (ExpBVSelGrOff)

The criterion ExpBVSelGrOff corresponds to the expected genetic level of selected grand-offspring that are produced by selected offspring of a mating. The motivation for the ExpBVSelGrOff criterion in contrast to the ProbSelGrOff criterion is, that a very low probability of either the first or the second term in the calculation of ProbSelGrOff causes it to be close to zero. For example, a close to zero probability for offspring to be selected will cause the entire term to be close to zero even if the conditional probability of grand-offspring to be selected is very high. By looking at the genetic level of selected grand-offspring as in ExpBVSelGrOff, this is avoided because the property of the offspring and grand-offspring generation are not combined multiplicatively, but additively. This is because the selection differential realized in the grand-offspring is added to the selection differential realized in the offspring. Equation (8) shows the calculation of the ExpBVSelGrOff criterion. For the calculation of ExpBVSelGrOff, we assumed that the same selection intensity is used across generations. In this study, 40 individuals were selected out of 4000 (1%) as described in the section ‘Breeding program’ later, which results in a selection intensity of 2.67. We also assumed the genetic variance in the population to remain approximately constant over generations. Additional file 1: Text S1 shows the derivation of Eq. (8).

8 $$ExpBVSelGrOff=0.25*\left({BV}_{sire}+{BV}_{dam}\right)+{i}_{p}*\left(0.5*\sqrt{{\sigma }_{FS}^{2}}+\sqrt{{\sigma }_{GO}^{2}}\right)$$

### Calculation of gametic Mendelian sampling variance of offspring

In this section, we present our approach to calculate the expected gametic MSV of offspring of a mating (${\overline{\sigma }}_{{gamMS}_{off}}^{2}$). Our approach combines methods published in plant breeding research and animal breeding research.

**Fig. 4:**
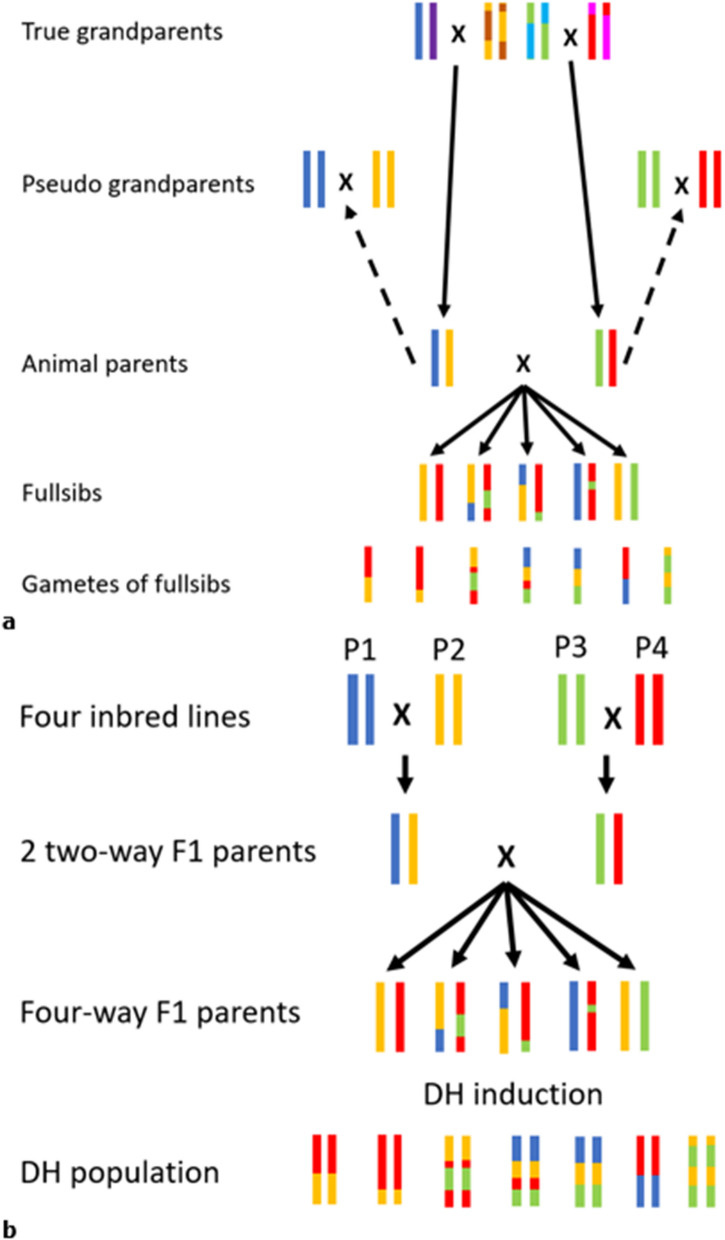
Transmission of chromosome segments in **a** an animal breeding setting and **b** a DH population based on four inbred founders considered by the approach by Allier et al. [9]. The parents to the genotypes labelled as “animal parents”, i.e., "true grandparents", in plot (**a**) are not unique to the “animal parents” because the mating of genotypes labelled as “pseudo grandparents” would result in the same parent genotypes. The “pseudo grandparents” are identical to the four inbred lines considered in the plant breeding setting in (**b**) and were considered to calculate the DH variance

Allier et al. [9] presented an analytical method to calculate the variance of double haploid (DH) lines created from four fully homozygous founder lines in a plant breeding setting. DH are individuals that are derived by doubling the chromosomes of the gametes of an individual to restore diploidy and produce fully homozygous lines. Thus, DH show the same linkage disequilibrium and allele frequencies as the gametes which are used to produce the DH. DH produced by four fully inbred lines are conceptually comparable to gametes produced by fullsibs in an animal breeding setting (see p. 93 in [23]). Figure 4 visualizes this similarity. By pretending that the grandparents in an animal breeding setting are fully homozygous for the chromosome that was inherited by the parent, these “pseudo grandparents” in animal breeding are identical to the actual inbred founder lines considered in the approach by Allier et al. [9] (Fig. 4).

To derive the expected gametic MSV of a random fullsib offspring (${\overline{\sigma }}_{{gamMS}_{off}}^{2}$), the variance among fullsibs (${\sigma }_{FS}^{2}$) needs to be subtracted from the variance of all DH (${\sigma }_{DH}^{2}$). This is because the variance of DH is not only influenced by the gametic MSV of the fullsibs but also by the variance of breeding values among the fullsibs. This difference then needs to be divided by 4 which is the factor by which variances on a DH level can be converted to variances on a gametic level. Thus, we developed Eq. (9) to calculate the expected gametic MSV of offspring individuals of a particular mating. Results from simulation are in agreement with the prediction based on Eq. (9) (results not shown). A proof is given in Appendix 1.

9 $${\overline{\sigma }}_{{gamMS}_{ off}}^{2}=\frac{\left({\sigma }_{DH}^{2}-\left({\sigma }_{{gametes}_{sire}}^{2}+{\sigma }_{{gametes}_{dam}}^{2}\right)\right)}{4}=\frac{\left({\sigma }_{DH}^{2}-{\sigma }_{FS}^{2}\right)}{4}$$

We calculated ${\sigma }_{DH}^{2}$ with the analytical equation presented by Allier et al. [9] for four-way DH populations. For the calculation of ${\sigma }_{{gametes}_{sire}}^{2}$ and ${\sigma }_{{gametes}_{dam}}^{2}$, we used the method presented by Musa and Reinsch [5] in their Equation (5).

### Selection and mate allocation

Both the ExpBVSelOff and the ProbSelOff criteria can be calculated using the properties of parents, i.e., their breeding values and their gametic MSV. Since high values in both of these properties will maximize ExpBVSelOff and ProbSelOff, the value of a mating does not depend much on the combined properties of both parents. In other words, a sire with a high breeding value and a high gametic MSV should always be preferred regardless of the properties of the dam it is mated to. That means that it is possible to express the probability to produce top offspring for a single animal instead of a mating. This is the idea behind Index5 of Bijma et al. [3], which aims at selecting animals with high breeding values and high gametic MSV. The benefit of expressing the probability to produce top genotypes for an animal instead of for a mating is that animals can be selected instead of pairs of animals.

Our newly developed criteria ExpBVSelGrOff and ProbSelGrOff are fundamentally different, as the value of a mating does depend on the combination of the parents. The parameter that is influenced by the combination of parents is the expected gametic MSV of offspring (${\overline{\sigma }}_{{gamMS}_{off}}^{2}$).

For selection, this means that animals need to be selected based on information that is expressed for pairs of animals. This is less straightforward than sorting animals based on their breeding value, or Index5 values, and simply selecting the best. Solving this optimization problem is not the focus of our study. To still be able to consider mating information in selection and mating, we separated the selection from the mate allocation problem and developed simple algorithms for each problem.

### Selection algorithm

In this section, we describe the selection algorithm that allows to select individuals based on values that are expressed for a potential mating, i.e., a pair of individuals.

We call the simple heuristic algorithm that we developed for selection the “remove least-liked algorithm” based on the fact that it removes the least liked animal one by one. The algorithm is made up of the following steps:

1. All animals are ranked for all potential mating partners based on the value calculated with the respective criterion.
2. The highest rank an individual ever got from the opposite sex is noted.
3. The individual with the lowest value for the highest rank (i.e. the “least liked” animal) is removed from the list of selection candidates.
4. Repeat until the desired number of males and females is selected.

**Fig. 5:**
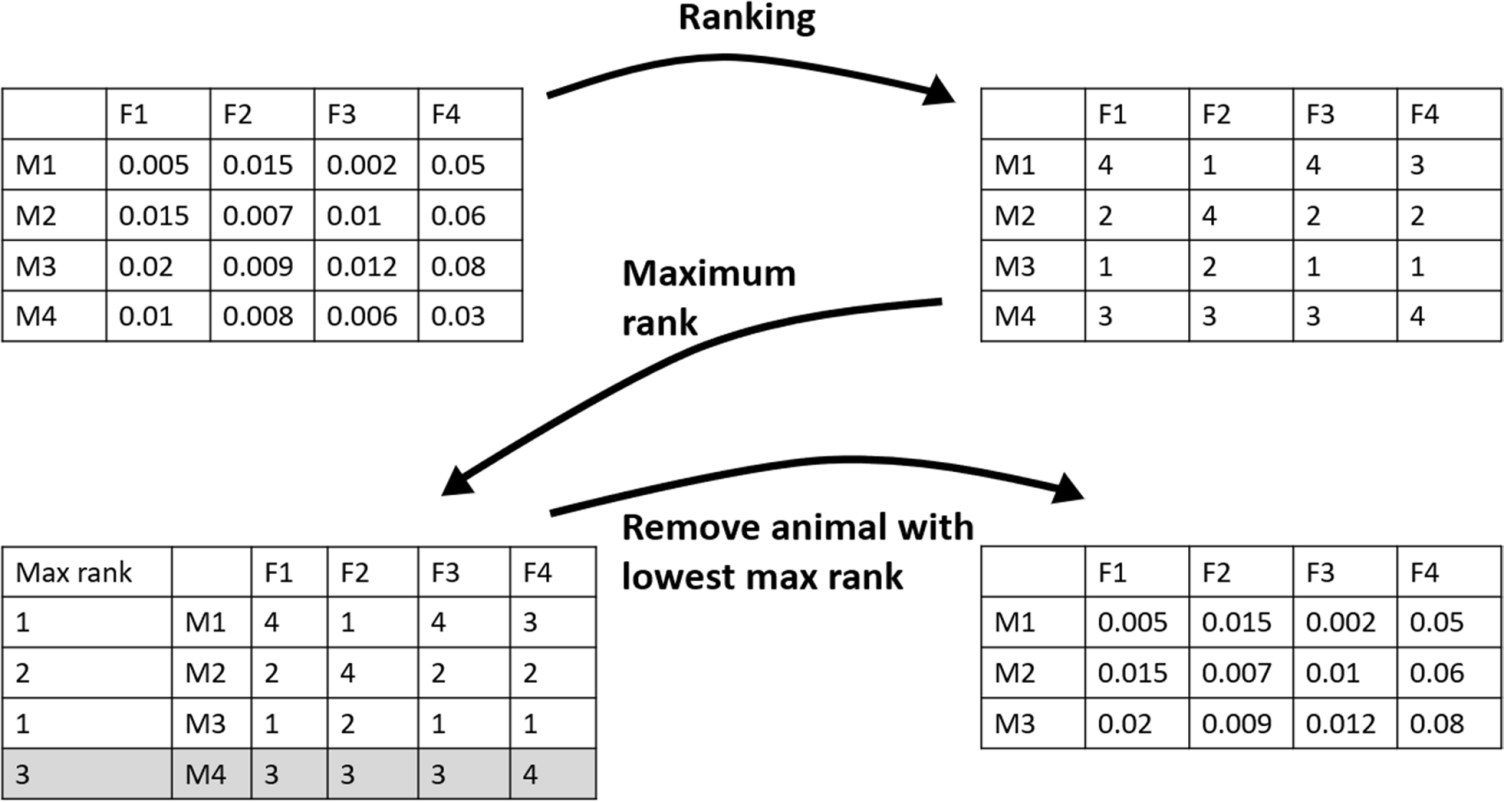
Schematic explanation of the remove least-liked algorithm. Only males (M) are ranked for each female (F) in this example. The individual highlighted in gray is the least-liked one and will be removed for the next iteration of the algorithm

Figure 5 shows a visual representation of the algorithm. First, a matrix is created that indicates the value for a pair of animals according to a certain selection criterion. Then, the males are ranked within each female. Next, the highest rank that a male has across all females is noted. The male with the lowest highest rank is removed and the algorithm starts from the beginning again. This process is repeated until the desired number of males is selected. For the sake of illustration, Fig. 5 considers that only males are selected, while the algorithm used in our study does consider both sexes simultaneously. This is done by removing the least liked animal regardless of sex if the number of animals to be selected of that sex has not been reached. We used this remove least-liked algorithm for selection when using the criteria ExpBVSelOff, ProbSelOff, ProbSelGrOff and ExpBVSelGrOff.

### Mate allocation algorithm

In this section, we describe the mate allocation algorithm that aims at allocating a mating partner in such a way that the partner for which the criterion value of the mating is the highest is chosen.

The mate allocation algorithm only requires that the contributions of each parent are predetermined and a restriction is set on how many offspring can be produced from a particular mating. The mate allocation algorithm was developed with the aim to have a chance for every animal to be mated to every selected animal of the opposite sex. The mate allocation algorithm is comprised of the following steps:

1. Sample a female randomly.
2. If the female has not reached the number of offspring it should produce, continue.
  Otherwise, go to step 1.
  - Pick the male that is most preferred by the female.
  - If the male has not yet reached its number of offspring it should produce, continue.
  - Otherwise, go to step 2a to pick the next best male.
  - If the maximum number of offspring allowed for this mating has not yet been reached, continue.
  - Otherwise, go to step 2a to pick the next best male.
3. Mate the selected female with the chosen male.
4. Go back to step 1 until the required number of matings has been achieved.

The algorithm requires to pick one of the sexes to draw from in step 1, and then use the other in step 2. This choice is unlikely to be reciprocal, i.e. the best matching male to mate with a particular female may not have this same female as its best matching mate. To account for these non-reciprocal choices, for half the number of offspring (2000), the mating partners for the females were chosen. For the other half, the mating partners for the males were chosen.

### Data simulation

We simulated a nucleus breeding population with recurrent selection with the simulation software MoBPS version 1.10.45 [25]. We followed the population set-up of Jibrila et al. [26] and simulated a species with 30 chromosomes each 100 centi Morgan long. We started by simulating a historical population to establish linkage disequilibrium among markers. This historical population involved 3000 generations of random mating, starting with a population size of 2500 males and 2500 females. The population size decreased linearly to 50 animals in generation 2997, and then increased linearly to 5000 animals in generation 3000.

After simulating the population history, about 15,000 markers of the initial 63,000 were still segregating. We placed 3000 purely additive QTL effects on randomly selected loci from a pool of loci with a minor allele frequency above 0.05, simply to ensure that enough effect loci are segregating. The QTL effects were drawn from a gamma distribution with a shape parameter of 0.4 in concordance with Jibrila et al. [26]. These settings resulted in a coefficient of variation of the standard deviation of gametic breeding values of 0.095 in generation 0 which is only slightly lower than the 0.10 value that Bijma et al. [3] found in dairy cattle based on data of Segelke et al. [7].

To prevent that the Bulmer effect impacts our results, we simulated an additional burn-in phase of five generations (3001 to 3005) with selection based on breeding values and random mating among the selected individuals. Afterwards, we simulated a breeding program for 21 generations (3006 to 3026) for each of the criteria for mating and selection.

In this study, we assumed that the genetic map of the species as well as the haplotypes, true QTL effects and thus the true breeding values of all animals are known without error.

### Breeding program

The population size in the breeding program was 4000 per generation. In every generation, 20 females and 20 males were selected based on the respective criteria. The selected parents contributed equally to the next generation, having 200 offspring each. Mating was restricted such that no more than 40 offspring were produced from any one mating. This ensured that every individual is mated to at least 25% of available selected partners of the opposite sex. The mate allocation algorithm was used when selection was based on the ExpBVSelOff, ProbSelOff, ExpBVSelGrOff and ProbSelGrOff criteria. For selection based on breeding values and Index5, mating was at random within the described restrictions.

In order to reduce computation time, we preselected 500 (25%) of all selection candidates per sex based on their breeding values. Bijma et al. [3] show in their Figure 2 that a preselection intensity as stringent as 10% does not have a negative effect when using Index5 values. To be on the safe side and also because we do not only select based on Index5 values, we opted to preselect 25% of all animals based on their breeding values. For these preselected candidates, the Index5 values, ExpBVSelOff and ProbSelOff were calculated and subsequently used for selection. In scenarios in which selection was based on the ProbSelGrOff and ExpBVSelGrOff criteria, a second preselection step was used in which four times more animals than needed for replacement were preselected from each sex based on their Index5 values (16% of all breeding-value-preselected-candidates). This second preselection step has the largest effect on computation time as the calculation of the expected offspring gametic MSV takes the longest time. The preselection intensity for this step was somewhat arbitrarily chosen.

The simulation and subsequent analyses were repeated 100 times for every selection criterion. All the code for this study was run in R version 4.1.2 on the high performance cluster annuna of Wageningen University & Research.

### Analyzed parameters

The following parameters were recorded every generation:

1. Average breeding value of the population relative to the average breeding value of the first generation in the breeding program (conventional genetic gain).
2. Average breeding value of the 20 best males and females relative to the average breeding value of the first generation in the breeding program (commercial genetic gain).
3. Average kinship level.
4. Bulmer-unimpacted true genetic variance.
5. Genic variance.
6. Number of beneficial alleles lost.

The average breeding values of the 20 best animals per sex represent the commercial genetic level, because semen or offspring of these animals can be sold to improve the genetic level of production animals. These best animals may not necessarily be selected themselves as parents to breed top genotypes in the next generation because they may not have a high gametic MSV or their offspring might not have a high gametic MSV.

To derive the Bulmer unimpacted genetic variance, we simulated four generations of random mating without selection in every generation and recorded the variance among breeding values in the fourth generation. The animals were discarded afterwards as this is just a pragmatic way of obtaining the genetic variance. We did this to be unbiased towards our criteria which reduce the variance of parent average breeding values less than selection based on breeding values does. As a consequence, variances of the breeding values of populations selected with our criteria are comparatively less impacted by the Bulmer effect and not accounting for the Bulmer effect when comparing variances would by design lead to declare our methods as best.

The genic variance was calculated as the sum over all loci of $2p\left(1-p\right){\alpha }^{2}$ with $p$ as the allele frequency of the allele and $\alpha$ as the additive effect of the allele. The genic variance was recorded in addition to the genetic variance because it is possible to increase the genetic variance just by changing the linkage disequilibrium structure without changing allele frequencies.

The reported kinship level was obtained as the average kinship level between 500 random animals for which kinship was calculated with the function kinship.emp.fast() of the MoBPS package [25]. This function traces all chromosome segments back to the founder generation and thus reports kinship as identity-by-descent. In this study, the founders are the animals at the beginning of the burn-in cycle (generation 3000). We only used 500 random animals in each generation for computation time reasons.

The degree of certainty of our results is shown with confidence intervals for the mean and the mean difference. Confidence intervals of the mean were calculated as the standard error of the mean of all observations per scenario multiplied by $\pm 1.96$ and added to the mean of all observations to obtain the 95% confidence interval. For the confidence interval of the mean difference, we calculated the standard error of the differences of the replicates of a particular scenario with the replicate of the breeding value scenario with the same seed. This reduces the variation induced by dependency between replicates based on populations generated with the same seed. In other words, the fact that different replicates have different genetic architectures is corrected for.

## Results

### Genetic gain

**Fig. 6:**
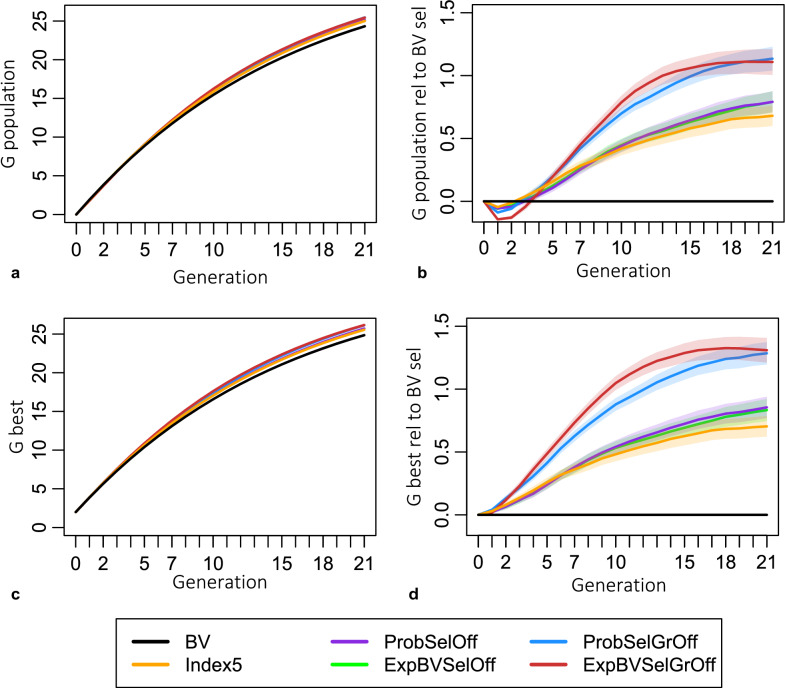
Evolution of the genetic level across generations. **a** Population average breeding value compared to generation 0 in units of genetic standard deviations of the base generation, **b** difference in population average breeding value compared to selection based on breeding values, **c** average breeding value of top 20 males and females compared to generation 0, **d** difference in breeding values of the best animals compared to selection based on breeding values. The shaded regions in plots (**a**) and (**c**) indicate the 95% confidence intervals of the means. The shaded regions in plots (**b**) and (**d**) indicate the 95% confidence interval of the mean difference of the genetic level achieved with a selection criterion compared to selection based on breeding values

All criteria considering gametic MSV resulted in significantly higher genetic gain after 21 generations compared to selection purely based on breeding values (plots a and b in Fig. 6). The short-term gain was slightly lower than with selection based on breeding values in terms of average breeding value of the population (Fig. 6, plot b). However, the commercial genetic gain, i.e., the breeding values of the best individuals of the population, was at no point compromised (Fig. 6, plot d). The criteria that look one generation ahead, ExpBVSelOff, ProbSelOff and Index5, resulted in, respectively, a 3.7%, 3.7% and 3.1% higher commercial genetic level compared to selection based on breeding values in generation 21. The criteria that look two generations ahead performed even better, and ExpBVSelGrOff and ProbSelGrOff resulted in 5.7% and 5.6% higher commercial genetic levels in generation 21 compared to selection based on breeding values. An overview of the performances in generations 1, 5 and 21 is in Table 3.

**Table 3:** Genetic gain compared to selection based on breeding values

	Conventional genetic gain			Commercial genetic gain		
	gen 1	gen 5	gen 21	gen 1	gen 5	gen 21
BV	0	0	0	0	0	0
ExpBVSelOff	− 3.0	1.4	3.3	1.3	2.9	3.7
ProbSelOff	− 2.8	1.2	3.3	1.2	2.9	3.7
Index5	− 2.3	1.8	2.8	1.6	3.1	3.1
ExpBVSelGrOff	− 7.2	2.2	4.6	1.0	5.8	5.7
ProbSelGrOff	− 4.5	2.2	4.7	2.1	4.9	5.6

Additional genetic gain compared to selection based on breeding values in percentage; calculated as $100\%\left(\frac{{\overline{BV} }_{criterion\_gen21}-{\overline{BV} }_{gen0}}{{\overline{BV} }_{BV\_gen21}-{\overline{BV} }_{gen0}}\right)-100\%$

### Genetic diversity

**Fig. 7:**
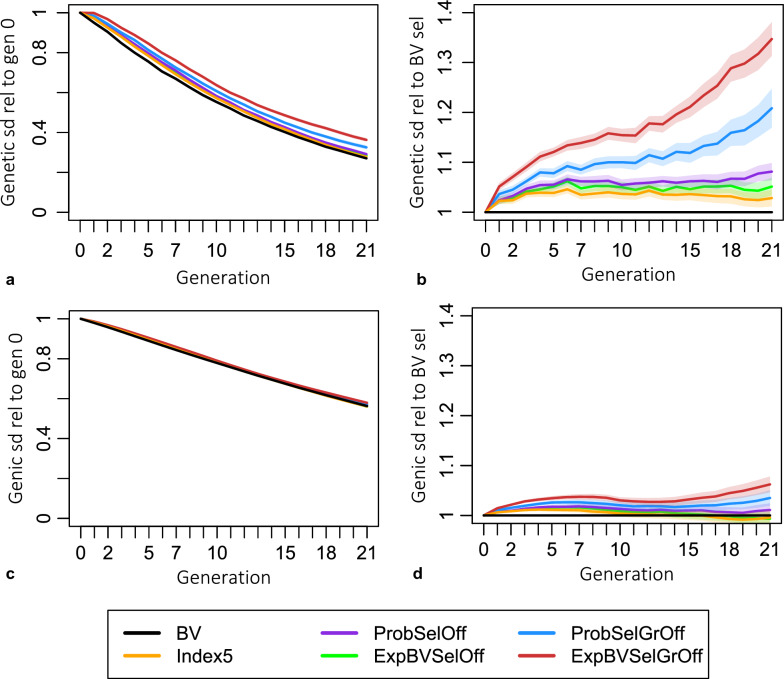
Evolution of genetic variation across generations. Genetic standard deviation (gSD) after four generations of random mating without selection (deemed unimpacted by the Bulmer effect) relative to gSD in generation 0 (**a**) and relative to gSD from breeding value selection schemes in the same generation (**b**). Genic SD relative to that in generation 0 (**c**) and to that from selection schemes based on breeding values (**d**). Values are the ratios obtained from the calculation of ($\frac{\sqrt{{varG}_{criterion\_gen21}}}{\sqrt{{varG}_{BV\_gen21}}}$)

All the methods using gametic MSV preserved significantly more genetic variance at any time point than truncation selection purely based on breeding values (Fig. 7, plot a), with ExpBVSelGrOff selection preserving the most. The same pattern is observed when comparing the genic variances with the only exception of ExpBVSelOff and Index5 which resulted in slightly lower genic variances in the last generations when comparing to selection based on breeding values (see plot b in Fig. 7 and Table 4). In any generation, criteria that use variances of descendants retain comparatively more genetic variance than genic variance compared to genetic and genic variances of populations selected based on breeding values (compare plot a and plot b in Fig. 7, and compare to genetic standard deviations for selection based on breeding values in Table 4; see Discussion).

**Table 4:** Maintenance of genetic variation relative to generation 0

	Genetic standard deviation			Genic standard deviation		
	Gen 1	Gen 5	Gen 21	Gen 1	Gen 5	Gen 21
BV	95.0	75.6	27.1	95.9	79.1	31.8
ExpBVSelOff	97.2	79.4	28.4	96.5	80.4	31.5
ProbSelOff	97.2	79.7	29.3	96.5	80.5	32.1
Index5	97.1	78.4	27.8	96.5	80.0	31.6
ExpBVSelGrOff	99.8	84.6	36.5	97.2	81.8	33.7
ProbSelGrOff	98.4	81.3	32.9	96.7	81.1	32.9

Percentage of genetic standard deviation and genic standard deviation retained by different selection criteria. Percentages are expressed relative to the first generation of the breeding program. Understand values as e.g., the genetic standard deviation in generation 5 was 79.4% as high as the genetic standard deviation in generation 0 when selecting based on ExpBVSelOff. Calculations follow the style of $100\mathrm{\%}\left(\frac{\sqrt{{varG}_{criterion\_gen21}}}{\sqrt{{varG}_{crterion\_gen0}}}\right)$

**Fig. 8:**
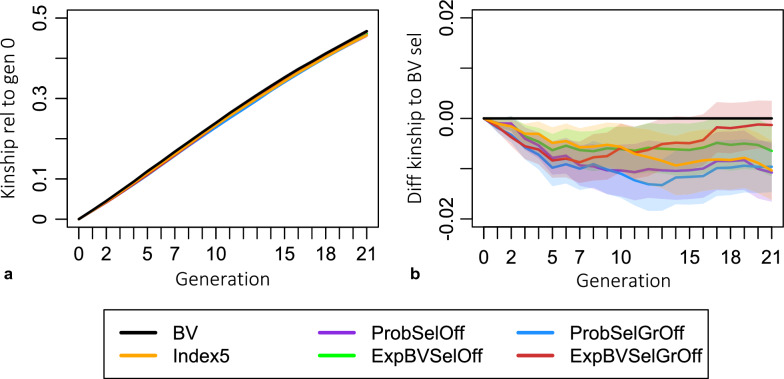
Evolution of average kinship levels across generations. Plot (**a**) shows the difference in kinship level to generation 0 and confidence intervals for the average kinship level. Plot (**b**) shows the absolute difference in kinship level compared to selection based on breeding values with confidence intervals for the mean difference

In addition to preserving more genetic and genic variance, all criteria using MSV also resulted in lower average kinship levels and lost less beneficial alleles (Table 5). Among all the criteria using MSV, the criteria that look two generations ahead resulted in the lowest kinship levels in generation 5. The average kinship achieved with ExpBVSelGrOff and ProbSelGrOff based selection was 7.0% and 8.2% lower than the average kinship realized with selection based on breeding values in generation 5, respectively (see Table 5). Interestingly, all the criteria using MSV resulted in much lower kinships in earlier generations than in later ones but never exceeded those of selection based on breeding values (see plot b in Fig. 8).

**Table 5:** Kinship and number of lost beneficial alleles relative to selection based on breeding values

	Average kinship			Number beneficial alleles lost		
	Gen 1	Gen 5	Gen 21	Gen1	Gen 5	Gen 21
BV	0	0	0	0	0	0
ExpBVSelOff	− 4.4	− 5.3	− 1.4	− 2.5	− 6.8	− 3.5
ProbSelOff	− 4.5	− 6.6	− 2.3	− 2.7	− 7.7	− 4.9
Index5	− 4.7	− 4.1	− 2.2	− 1.2	− 5.0	− 4.0
ExpBVSelGrOff	− 7.3	− 7.0	− 0.3	0.3	− 12.4	− 4.0
ProbSelGrOff	− 8.4	− 8.2	− 2.1	− 1.7	− 9.6	− 5.6

Percentage difference in kinship level and number of lost beneficial alleles. Negative values need to be understood as benefit, i.e., less kinship or less lost beneficial alleles. Values have to be interpreted as e.g., ExpBVSelOff based selection lost 6.8% less beneficial alleles than breeding value based selection after 5 generations. Calculations follow the style of $100\%\left(\frac{{\overline{kinship} }_{criterion\_gen21}-{\overline{kinship} }_{gen0}}{{\overline{kinship} }_{BV\_gen21}-{\overline{kinship} }_{gen0}}\right)-100\%$

## Discussion

We investigated the use of selection criteria that use MSV in a recurrent selection breeding scheme to accelerate genetic progress. Our newly developed criterion ExpBVSelGrOff performed the best. It achieved the highest genetic gains without compromising genetic diversity. We attribute this benefit to the longer planning horizon used for ExpBVSelGrOff in comparison to previously proposed methods that only optimize one generation ahead.

### Distinction between conventional and commercial gain

The application of MSV-considering criteria is only beneficial if it is acceptable that the population average breeding value increases less in the short term compared to selection based on breeding values. This is because Index5, ExpBVSelOff and ProbSelOff focus on maximizing the genetic level of the best animals in the offspring generation, which is equivalent to the population average breeding value in the grand-offspring generation. The criteria ExpBVSelGrOff and ProbSelGrOff focus on maximizing genetic gain even one more generation ahead. Thus, by definition, the population average breeding value up to that focal generation is compromised (Table 3). Intuitively speaking from a maximization point of view, considering any additional parameter, like the MSV, next to breeding values causes the average breeding value of the group of selected candidates to be lower than if selection was based on breeding values alone. That is why the conventional genetic gain is lower in early generations (Fig. 6 plot b and Table 3 the first column). However, in breeding programs in which the genetic material is disseminated from the best individuals, such as artificial insemination boars or bulls, the variance and the mean of the breeding value distribution are both relevant for commercial genetic gain, and commercial genetic gain was never compromised (Table 3 and Fig. 6).

### Why are selection thresholds different for probability and genetic gain criteria?

During the development of this study, we had first developed the ProbSelGrOff criterion. Later, we thought that the ProbSelGrOff criterion has the disadvantage that the probability of selecting good offspring and the probability of selecting good grand-offspring are combined multiplicatively. If one of these probabilities is zero, or very close to zero, the entire product will be zero. This happens for example when two unrelated but highly performing parents both have a low gametic MSV, e.g. because they are highly inbred. If those unrelated parents carry different beneficial haplotypes, then mating them would result in a low variance of breeding values of their offspring (F1) but these offspring will themselves have a high gametic MSV. Thus, the conditional probability that grand-offspring (F2) will be selected will be high (see Eq. (7)).

In an attempt to solve this problem, we later designed the ExpBVSelGrOff criterion in which the expected gain realized in offspring is additively combined with the gain in grand-offspring. By shifting the focus from the probability to the genetic merit, the question arises which absolute truncation selection points should be used for the offspring and grand-offspring. Using the same absolute truncation selection points as for the ProbSelGrOff criterion, i.e., the absolute truncation selection points are the same for all families, may seem, at first, the intuitively best choice. However, using the same truncation points means that the offspring and grand-offspring of different matings have different probabilities to show a breeding value larger than the truncation point. However, when the criterion is the expected average genetic level of selected offspring or grand-offspring using the same truncation selection points for all families, the differences in probabilities to actually select these top offspring or grand-offspring are ignored.

The following example illustrates this problem. Let us consider the counterpart criteria that only look one generation ahead, i.e., ProbSelOff and ExpBVSelOff. If the selection intensities in the calculation of the ExpBVSelOff criterion were chosen so that they correspond to the same absolute truncation selection point as considered in the ProbSelOff criterion, then the predicted genetic merit does not reflect the likelihood, i.e., the risk, that an offspring with such a high breeding value will be produced. This would result in different decisions than those made by ProbSelOff as visualized in Additional file 2: Fig. S1. We tried using the ExpBVSelOff criterion with the same absolute truncation selection point for all matings during the development of this study but we always observed worse performances (results not shown). Thus, we continued with using the same selection intensity per mating for the ExpBVSelOff and ExpBVSelGrOff criteria, i.e., we assumed that the same fraction of offspring and grand-offspring of every mating would be selected.

### Difference to the usefulness criterion

Although the equation of the ExpBVSelOff criterion is in essence identical to the usefulness criterion [16], our motivation for the choice of selection intensity to be used for ExpBVSelOff and ExpBVSelGrOff differs from the one used by Schnell and Utz [16] for variety development. The definition of the usefulness of a cross according to Table 1 of Schnell and Utz [16] is the expected performance of the best genotype that can be realized from a cross between two particular parents. This means that they expected that one offspring line, namely the best one, is selected out of all the lines produced by a cross. In other words, the choice of selection intensity is based on the expected fullsib family size. While this may seem reasonable at first for crop species where tens or hundreds of offspring genotypes can be obtained from a cross, this concept does not work well for typical livestock species that have a much lower reproduction factor. The reasoning for the choice of selection intensity in the original definition of the usefulness criterion seems valid if the breeder can only select one cross to make and out of which to select a variety. However, breeders typically make more than just one cross and thus the top genotype that will be marketed as the new variety may come from any cross. Considering the same selection intensity, with which the population would be selected, for all matings as we propose, solves this issue.

### Influence of population size

In this proof of concept study, we selected 20 males and 20 females every generation to generate 4000 selection candidates for the next generation meaning every female needs to produce 200 offspring. These numbers were chosen so that we could apply a high selection intensity to better see the effects. This is motivated by the Table 4 in Bijma et al. [3] who show that the benefit of including the gametic MSV in selection decisions increases as the proportion of selected individuals decreases. However, the numbers used in our simulation exceed the female reproductive capacity of typical livestock species. In addition, females are typically mated to one, or only a few males in the case of embryo transfer, whereas we allowed mating to all available males. These may be unrealistic assumptions for current animal breeding practice. Accounting for these biological limitations was not the focus of the simulation study. We do not expect a different ranking of selection criteria had we accounted for these biological limitations because the benefit of planning further ahead stems from finding haplotypes that are more dissimilar in order to select individuals, or offspring of matings, with higher gametic MSV.

The number of offspring per mating has no effect on the benefit of the selection criteria. This can be made intuitive by considering the ProbSelOff criterion but the explanation also holds for all the other criteria. Although this probability that a random offspring individual shows a breeding value larger than the selection threshold is expressed for a potential mating, one can easily think that it is the value of a random offspring of that mating for which genotypic or phenotypic information has not been collected. This probability does not change whether the offspring individual has 50 or 0 siblings. Thus, large fullsib groups are not required for the success of employing MSV-considering criteria in breeding programs.

We used our mate allocation algorithm to assign mates selected based on ExpBVSelOff, ProbSelOff, ExpBVSelGrOff and ProbSelGrOff whereas mating of individuals selected based on breeding values and Index5 was random. The mate plan solutions based on ExpBVSelOff and ProbSelOff are also essentially random ones because these criteria are only influenced by the gametic MSV of the parents, which are independent from each other. In contrast, mate plan solutions for ExpBVSelGrOff and ProbSelGrOff are less like random solutions because the values of these criteria depend on the gametic MSV of offspring of the mating, i.e., there is interaction between parents. Connecting to the quantitative genetic prediction of gametic MSV of offspring based on relationships, this means that the mate plan solutions for these two criteria are more similar to mating the least related animals. It is known that minimum coancestry mating compared to random mating causes lower homozygosity in progeny, which in turn increases genetic gain slightly [27] so one might think that the added benefit stems from the mating technique alone. During the development of this study, we also investigated the use of our new criteria with and without mate allocation and indeed observed a slight beneficial effect when using our mate allocation strategy (results not shown). However, this effect was smaller than the choice of selection criterion had on genetic gain, hinting that selection, and not mating, is the most critical factor in increasing the response to selection. We included our mate allocation strategy in the presented study to demonstrate the full potential of the considered criteria. Nevertheless, even in situations where it is impractical to design matings of all selected animals, most of the potential gain of using the MSV based criteria can be obtained.

### Effects on diversity

Criteria that use the MSV generally maintained more genetic diversity than the selection scheme that based decisions on breeding values only. This means that more genetic gain can be achieved without harming diversity. We looked at different measures of genetic diversity because they all describe different aspects of diversity. In our opinion, the most relevant metric for practical breeders is the genetic variance that is not affected by the Bulmer effect. Our newly developed criteria maintained the most genetic variance throughout the breeding program, meaning that they show the potential to generate the largest genetic gains. Although the genic variance maintained by ExpBVSelGrOff and ProbSelGrOff was also larger than that of selection based on breeding values, the superiority of our criteria was relatively lower for the genic variance than for the genetic variance (compare plots a and b in Fig. 7). When comparing the percentage of maintained genetic and genic standard deviation as presented in Table 4, it can be seen that, when selection is based on breeding values, relatively more genic standard deviation than genetic standard deviation was maintained. The same applies to criteria looking one generation ahead from generation 5 onwards. Interestingly, when selecting with our two new criteria that look two generations ahead, relatively more genetic standard deviation than genic standard deviation was maintained. Linkage is ignored in the calculation of the genic variance, whereas it is considered in the calculation of the genetic variance. Therefore, the differences in results for the genic and genetic variance reflect differences in the linkage disequilibrium between QTL. More precisely, when applying directional selection based on the breeding values, the linkage disequilibrium between beneficial alleles is well-known to be negative, which results in negative covariance [28]. In our simulation experiment, we see that the difference between genetic and genic variance gets smaller, as more generations a criterion looks ahead. The ExpBVSelGrOff criterion even showed a larger genetic variance than genic variance after the four generations of random mating. This means that the ExpBVSelGrOff criterion has created positive linkage disequilibrium between beneficial alleles. The reason for why the linkage disequilibrium becomes less negative, as more generations are planned ahead, is because the focus of the criteria is not only on breeding values but also on gametic MSV and gametic MSV in the offspring. Generally, the more heterozygous an animal is, the higher its gametic MSV. However, in real life and in this study, the alleles are linked on chromosomes and may vary in their effect size. Thus, it is not actually the heterozygosity that predicts gametic MSV, but the joint effect of heterozygosity, linkage and effect size. In simple terms, the higher the gametic MSV is, the larger the difference between the breeding values of the two haplotypes of an animal is. And this difference becomes the larger as more beneficial alleles are in coupling phase. Since haplotypes can be passed on to the next generation, i.e., are heritable, using genetic variances of descendants in selection decisions results in relatively less negative covariance between beneficial alleles which increases genetic variance.

Another metric aiming at describing potentially useful diversity is the number of beneficial alleles that have been lost. If beneficial alleles are lost from the population, it is impossible to bring them back. Thus, the more beneficial alleles have been lost, the lower the genetic value is for the theoretically optimal genotype in which all remaining beneficial alleles are combined. All criteria using MSV lost fewer beneficial alleles compared to selection based on breeding values (see Table 5). ExpBVSelGrOff was the best criterion in generation 5 but was outperformed by ProbSelOff and ProbSelGrOff in generation 21 suggesting that ExpBVSelGrOff may not be the best criterion in this respect. However, when combining with observations from the genic variance (plot b, Fig. 7), ExpBVSelGrOff always showed the largest genic variance. The genic variance is larger, as more beneficial alleles are segregating, the effect size of segregating alleles is larger and the allele frequencies are closer to 50%. Thus, we hypothesize that the genic variance of ExpBVSelGrOff was larger because this criterion either manages to make better trade-off decisions to lose rather small effect beneficial alleles, or it manages to move allele frequencies to 50% faster.

Lastly, we also investigated the development of kinship levels over generations. Contrary to the aforementioned metrics, mean kinship describes the diversity at all loci including neutral loci. The diversity expressed by the kinship can thus be understood as the diversity of traits that may be included in the breeding goal in the future [29]. The average kinship level is equivalent to the average inbreeding levels in the next generation under random mating. Higher inbreeding levels pose a higher risk of inbreeding depression, and higher kinship levels indicate loss in neutral diversity. We showed that all the MSV-considering criteria resulted in lower kinship levels than selection based on breeding values (Table 5 and Fig. 8). This is not surprising, considering that the quantitative genetic expectation of the gametic MSV of an individual is proportional to its inbreeding level under the infinitesimal model; $gamMSV=\left(1-F\right)* {\sigma }_{A}^{2}*0.25$ [6]. Hence, criteria that consider the MSV should be more likely to lead to the selection of individuals, or matings, that are less inbred and less related.

Our findings are in line with results of other simulation studies in which either the Index5 [5] or the usefulness criterion [21, 22, 30] was used for selection in comparison to selection based on breeding values. Musa and Reinsch [5] also report lower inbreeding levels which is in line with the lower kinship levels reported here. All studies report higher genetic gain and smaller numbers of fixed QTL alleles compared to selection based on breeding values, which is intriguing given that Neyhart et al. [30] also showed faster changes in allele frequencies. An explanation for a faster rate in frequency change without loss of variants may be that allele frequencies are pushed faster towards 50%. In comparison to selection based on breeding values, larger genetic variances [5, 22, 30] as well as larger genic variances have been reported [22]. In their Figure 6, Allier et al. [22] show that the ratio of genetic variance over genic variance over generations reflects the Bulmer effect. The Bulmer effect causes a slight negative covariance between beneficial loci (see p. 202 in [23]), which consequently causes the ratio to be lower than 1. However, they show that when selection is based on the usefulness criterion, this ratio increases over time and even exceeds 1 after 50 generations. This further supports our hypothesis discussed above that the linkage disequilibrium between beneficial loci, and thus the covariance, become less negative, or even positive, when the gametic MSV is included in selection decisions.

Increasing genetic diversity when including the MSV in selection is a welcome side-effect, although it was not the direct objective. Using the MSV in selection is rather like projecting the current diversity of the population into gain in the future. Or in other words, for these MSV-considering criteria, diversity is a necessity that is required for the goal of maximizing genetic gain.

### Estimating MSV

For the proof of concept and for the sake of simplicity, we assumed that all properties necessary for using the MSV are known without error. In reality, the predicted gametic variance will largely depend on the phasing quality and accuracy with which an effect is estimated. Moderate to high correlations for various traits between the predicted gametic Mendelian standard deviation and real variation of progeny breeding values have been reported for French and German Holstein dairy cattle [7, 31]. Traits for which variances can be predicted with reasonably high accuracy show greater promise to be used in MSV-considering criteria. Further research is needed to validate the performance of our suggested models if haplotypes and single nucleotide polymorphism effects are estimated in animal breeding programs.

As discussed in this study before, the gametic MSV of an individual can be predicted based on its inbreeding level when assuming the infinitesimal model. However, very low correlations (− 0.1 to − 0.2 based on genomic inbreeding) have been found in German Holstein cattle when trying to predict the gametic MSV based on inbreeding levels [7]. Similar results have been found in French Holstein cattle [8]. For comparison, in our simulation study, the correlation between identity-by-descent homozygosity level and the gametic MSV was approximately between − 0.2 and − 0.3 (results not shown). These findings show that the inbreeding level is a poor predictor of the gametic MSV, and suggests that differences in linkage and allele effect sizes appear to be the more significant factors that influence MSV.

### Use in plant breeding

We presented this study from an animal breeding perspective. All tested criteria, including ExpBVSelGrOff, can also be used in plant breeding programs. For example, in rapid cycling plant breeding schemes, where genetic gain is accelerated by drastically decreasing the generation interval [1, 32]. This is made possible by the use of genomic estimated breeding values which allow selection without field testing. In rapid cycling, no recombinant inbred lines or DH are produced for further population improvement. Thus, individual plants in a rapid cycling scheme are not fully homozygous and are thus directly comparable to individuals in animal breeding programs.

More classical breeding schemes with recombinant inbred lines or DH may also apply our criterion if selection is applied in an intermediate step, such as in the 4-way F1 parents as shown in Fig. 4, or when the final generation is selfed or DH are induced instead of being crossed to the population. In these cases, the required genetic variance terms simply need to be changed in the equations.

### Further development and other work

Further work could focus on extending our presented approach to consider even more generations, i.e., extending the planning horizon. To do so, analytical equations need to be developed to calculate the expected gametic MSV in great-grand-offspring, great-great-grand-offspring or more. While this is theoretically possible, the number of founders to consider in these calculations is growing exponentially with every additional generation. Furthermore, the number of combinations even in the same set of founders increases, or in other words, the question of who is mated to who and whose offspring is mated to who is becoming increasingly more complex with more generations. These combinatoric considerations are not negligible as they can influence the gametic MSV of their descendants, and pose a challenge to develop efficient selection algorithms. The focus of our study was on the development of new criteria rather than on well-performing selection and mating algorithms. Hence, our algorithms should by no means be understood as the perfect solution for selecting with ExpBVSelGrOff and ProbSelGrOff.

Our criteria are not designed to manage overall diversity, e.g. to preserve alleles with gene actions that are not known or alleles that affect a trait that may be of interest in the future. Our criteria were designed to maximize genetic gain in selected individuals in two generations ahead. The most established tool to manage overall diversity and genetic gains simultaneously is optimum contribution selection (OCS) [4, 33]. While the combination of Index5 with OCS is straightforward because Index5 expresses a quality for a single individual, further investigation is needed to elucidate whether and how the ExpBVSelGrOff criterion and OCS can complement each other. A potential approach may be to optimize the contributions of matings, i.e., the number of offspring per mating, instead of optimizing contributions of individuals. Optimization techniques have already been implemented and reported that can jointly optimize mate allocation and genetic contributions [21, 22, 34–38].

In contrast to our quantitative genetically motivated criteria, other techniques for decision making for selection and mating from the field of operations research have been proposed in the literature [39–41]. The most notable development is the look-ahead selection (LAS) approach presented by Moeinizade et al. [41]. LAS aims at solving the problem of selection, mating and contribution of parents simultaneously to achieve maximum gain within a given timeframe. LAS results in a mate plan which suggests selfing of the best individual in the second to last generation as reported by Zhang and Wang [39]. Equivalently, it would suggest mating the best dam to the best sire in an animal breeding setting. Zhang and Wang [39] present an improved LAS approach that replaces the need for a fixed timeframe by using a sliding window approach. The authors report that a window length of 3, i.e., maximizing genetic gain three generations ahead, performed best in their study for long-term gain. Maximizing genetic gain three generations ahead is equivalent to maximizing the average breeding value of the great-grand-offspring generation which is identical to what our criteria ExpBVSelGrOff and ProbSelGrOff aim at. Since selected grand-offspring are used as parents for great-grand-offspring, our criteria look the same number of generations ahead as the planning horizon in Zhang and Wang [39]. Zhang and Wang [39] note that the window length of 3 may only be optimal in the specific breeding scheme tested in their study and different window lengths may be optimal in other situations. It should also be noted that several more aspects are considered at the selection and mating step in the method by Zhang and Wang [39] which we did not consider. Thus, the results of our study are not directly comparable to theirs.

## Conclusions

We compared the use of selection criteria that consider the gametic MSV of selection candidates and the gametic MSV of offspring in addition to breeding values, to increase the chance of breeding top individuals. All criteria generally performed better than selection based on breeding values in terms of genetic gain and genetic diversity. We found that our criteria which plan two generations ahead, performed better with regards to genetic gain and maintaining useful variation than selection based on breeding values and the criteria that plan one generation ahead as evidenced in the larger retained additive genetic variance. Application of our criteria comes without additional cost for current genomic breeding programs except for additional computations. In this study for the proof of concept, we assumed that the true QTL effects, genetic map and the haplotypes of all animals are known without error.

### Appendix 1: Calculation of gametic Mendelian sampling variance of offspring

To derive the gametic Mendelian sampling variance of the offspring of a particular mating, we used Eq. (9). The expected within-line variance can be calculated with Eq. (10) according to Falconer and Mackay [23] p. 265 where ${V}_{W}$ is the within-line variance, ${V}_{G}$ is the genetic variance in the base population, $F$ is the inbreeding coefficient of the members of the line and $f$ is the coefficient of coancestry of individuals in the same line. The lines in our case are all offspring produced by the parents shown in Fig. 4 or produced by individuals E and F in Fig. 9. 

Fig. 9 Pedigree as considered in Fig. 4. The subscript “_dh” indicates that the individual is a double haploid. Individuals ”A_dh”, ”B_dh”, ”C_dh” and ”D_dh” are unrelated founders

Figure 9 shows the pedigree of individuals as shown in plot b of Fig. 4. An intuitive explanation of Eq. (10) is that the variance increases with higher levels of inbreeding because a larger fraction of the members of the line are homozygous for one of the alleles, i.e., more individuals have extreme breeding values than expected under random mating. The definition of the coefficient of coancestry is the probability that a randomly chosen allele in an individual is identical-by-descent to a randomly chosen allele at the same locus in the other individual (see p. 85 in [23]). Thus, the higher the coancestry coefficient is, the more similar are the allele frequencies in two individuals. This results in more similar breeding values of two individuals which reduces the genetic variance. For a complete description, see Falconer and Mackay [23].

10 $${V}_{W}=\left(1+F-2f\right){V}_{G}.$$

The variance of DH progeny produced by a single individual can be calculated with Eq. (10) as this variance is identical to the within-line variance. For DH produced by a single non-inbred individual, this variance is 1 ${V}_{G}$:

$${V}_{1FS:DH}=\left(1+1-2*0.5\right){V}_{G}=\left(1+1-1\right){V}_{G}=1{V}_{G}.$$

This is identical to the description of Dempfle [6] who presents a formula to calculate the gametic Mendelian sampling variance of an individual based on its inbreeding coefficient with ${V}_{gametes}=\left(1-F\right)*\frac{{V}_{G}}{4}$ where  $F$ is the inbreeding coefficient of the individual. For a non-inbred individual, the gametic variance is ${0.25V}_{G}$. This is different by a factor of 4 to the variance if DH were produced based on one individual. This factor is caused by gametes being haploid and DH being diploid.

The variance of full-sibs as shown in plot a of Fig. 4 and represented by G and H in Fig. 9 is 0.5 ${V}_{G}$:

$${V}_{FS}=\left(1+0-2*0.25\right){V}_{G}=\left(1+0-0.5\right){V}_{G}=0.5{V}_{G}.$$

The variance of the breeding values of double haploids produced by all full-sibs as shown in plot b of Fig. 4 and represented by I_dh and J_dh in Fig. 9 is 1.5 ${V}_{G}$:

$${V}_{allFS:DH}=\left(1+1-2*0.25\right){V}_{G}=\left(1+1-0.5\right){V}_{G}=1.5{V}_{G}.$$

The variance among full-sibs and the variance of double haploids produced by all full-sibs can be used to calculate the variance of DH offspring produced by a single full-sib offspring. Note that this is identical to the numerator of Eq. (9) which is ${(\sigma }_{DH}^{2}-{\sigma }_{FS}^{2})$:

$${V}_{1FS:DH}=\left(1+{F}_{allFS:DH}-2*{f}_{allFS:DH}\right){V}_{G}-(1+{F}_{FS}-2*{f}_{FS}) {V}_{G}.$$

which can be rewritten as:

$${V}_{1FS:DH}=\left(1-1+{F}_{allFS:DH}-{F}_{FS}-2*{f}_{allFS:DH}-\left(-2*{f}_{FS}\right)\right){V}_{G}.$$

The coefficient of coancestry for DH progeny produced by full-sibs ($${f}_{allFS:DH})$$, such as I_dh and J_dh, is 0.25. This is identical to the coefficient of coancestry of the full-sibs ($${f}_{FS})$$. The inbreeding coefficient $${F}_{allFS:DH}$$ for double haploids is 1 and the inbreeding coefficient of full-sibs $${F}_{FS}$$ is 0 as the parents are unrelated. Inserting these values gives a variance of 1 $${V}_{G}$$:

$${{V}_{1FS:DH}=(1-1+1-0-2*0.25-\left(-2*0.25\right){)V}_{G}=(0+1-0.5+0.5{)V}_{G}=1{V}_{G}}.$$

This is equivalent to the result of the calculation above for the variance of DH offspring produced by one individual when using Eq. (10) directly. Thus, the numerator of our Eq. (9) is correct. The denominator just follows from the fact that 4 is the factor with which to transform variances on a DH level to gametic variances. The numerator (${V}_{1FS:DH}$) cannot be calculated analytically based on allele substitution effects and linkage directly since no formula has been presented yet. Thus, we combined two published approaches for ${V}_{allFS:DH}$ (${\sigma }_{DH}^{2}$) and ${V}_{FS}$ (${\sigma }_{FS}^{2}$) by using the methods presented by Allier et al. [9] and Musa and Reinsch [5], respectively.

### Supplementary Information

**Additional file 1: Text S1.** Detailed explanation for the derivation of the ExpBVSelGrOff criterion.

**Additional file 2: Figure S1.** Differences in the decisions made for selection based on the ProbSelOff and ExpBVSelOff criteria.

## Data Availability

The datasets generated and/or analysed during the current study are not publicly available due to current investigation of application on funding partners’ breeding programs but are available from the corresponding author on reasonable request.

## Appendix 2: Calculation of the selection intensity and normalized truncation selection point for ProbSelGrOff criterion

When using criterion ProbSelGrOff, the normalized truncation selection point $x$, the selection intensity $i$ and thus the variance reduction coefficient $k$ are different for every mating. They depend on the parent average breeding value, the variance among fullsib offspring and the absolute truncation selection point.

The normalized truncation selection point can be calculated in the following way:

$$x=\frac{{{\tau }_{p}}_{O}-\left(\frac{{BV}_{sire}+{BV}_{dam}}{2}\right)}{\sqrt{{\sigma }_{FS}^{2}}}.$$

To calculate the selection intensity, the density at the normalized selection point $x$ is needed. The density function of the standard normal distribution is the following:

$$\varphi \left(x\right)=\frac{1}{\sqrt{2\pi }}{e}^{-0.5{x}^{2}}.$$

The selection intensity that applies to offspring of a particular mating is:

$$i=\frac{\varphi \left(x\right)}{ProbSelOff}.$$

## References

[1] Gaynor RC, Gorjanc G, Bentley AR, Ober ES, Howell P, Jackson R (2017). A two-part strategy for using genomic selection to develop inbred lines. Crop Sci.

[2] Santos DJA, Cole JB, Lawlor TJ, VanRaden PM, Tonhati H, Ma L (2019). Variance of gametic diversity and its application in selection programs. J Dairy Sci.

[3] Bijma P, Wientjes YCJ, Calus MPL (2020). Breeding top genotypes and accelerating response to recurrent selection by selecting parents with greater gametic variance. Genetics.

[4] Wellmann R, Bennewitz J (2019). Key genetic parameters for population management. Front Genet.

[5] Musa AA, Reinsch N (2023). A similarity matrix for preserving haplotype diversity among parents in genomic selection. bioRxiv..

[6] Dempfle L, Gianola D, Hammond K (1990). Problems in the use of the relationship matrix in animal breeding. Advances in statistical methods for genetic improvement of livestock.

[7] Segelke D, Reinhardt F, Liu Z, Thaller G (2014). Prediction of expected genetic variation within groups of offspring for innovative mating schemes. Genet Sel Evol.

[8] Hozé C, Baur A, Fritz S, Boichard D. Prediction of gametic variance and its use in breeding programs. In: Proceedings of the 71st Annual Meeting of the European Federation of Animal Science; 1–4 December 2020; Virtual Meeting. 2020. https://www.youtube.com/watch?v=pVHVtnkU8uQ. Accessed 13 Feb 2023.

[9] Allier A, Moreau L, Charcosset A, Teyssèdre S, Lehermeier C (2019). Usefulness criterion and post-selection parental contributions in multi-parental crosses: application to polygenic trait introgression. G3 (Bethesda)..

[10] Bonk S, Reichelt M, Teuscher F, Segelke D, Reinsch N (2016). Mendelian sampling covariability of marker effects and genetic values. Genet Sel Evol.

[11] Lehermeier C, Teyssèdre S, Schon CC (2017). Genetic gain increases by applying the usefulness criterion with improved variance prediction in selection of crosses. Genetics.

[12] Osthushenrich T, Frisch M, Herzog E (2017). Genomic selection of crossing partners on basis of the expected mean and variance of their derived lines. PLoS One..

[13] Zhong S, Jannink J-L (2007). Using quantitative trait loci results to discriminate among crosses on the basis of their progeny mean and variance. Genetics.

[14] Wolfe MD, Chan AW, Kulakow P, Rabbi I, Jannink J-L (2021). Genomic mating in outbred species: predicting cross usefulness with additive and total genetic covariance matrices. Genetics.

[15] Mohammadi M, Tiede T, Smith KP (2015). Popvar: a genome-wide procedure for predicting genetic variance and correlated response in biparental breeding populations. Crop Sci.

[16] Schnell F, Utz H. Bericht über die arbeitstagung der vereinigung österreichischer pflanzenzüchter. Gumpenstein: BAL Gumpenstein. 1975:243–8.

[17] Bernardo R (2014). Genomewide selection of parental inbreds: classes of loci and virtual biparental populations. Crop Sci.

[18] Beckett TJ, Rocheford TR, Mohammadi M (2019). Reimagining maize inbred potential: identifying breeding crosses using genetic variance of simulated progeny. Crop Sci.

[19] Michel S, Löschenberger F, Moreno-Amores J, Ametz C, Sparry E, Abel E (2022). Balancing selection gain and genetic diversity in the genomic planning of crosses. Plant Breed.

[20] Abed A, Belzile F (2019). Exploring the realm of possibilities: trying to predict promising crosses and successful offspring through genomic mating in barley. Crop Breed Genet Genomics.

[21] Sanchez D, Sadoun SB, Mary-Huard T, Allier A, Moreau L, Charcosset A (2023). Improving the use of plant genetic resources to sustain breeding programs' efficiency. Proc Natl Acad Sci USA.

[22] Allier A, Lehermeier C, Charcosset A, Moreau L, Teyssedre S (2019). Improving short- and long-term genetic gain by accounting for within-family variance in optimal cross-selection. Front Genet.

[23] Falconer DS, Mackay TFC (1996). Introduction to quantitative genetics.

[24] Bulmer MG (1971). The effect of selection on genetic variability. Am Nat.

[25] Pook T, Schlather M, Simianer H (2020). Mobps—modular breeding program simulator. G3 (Bethesda)..

[26] Jibrila I, Ten Napel J, Vandenplas J, Veerkamp RF, Calus MPL (2020). Investigating the impact of preselection on subsequent single-step genomic blup evaluation of preselected animals. Genet Sel Evol.

[27] Sonesson AK, Meuwissen TH (2000). Mating schemes for optimum contribution selection with constrained rates of inbreeding. Genet Sel Evol.

[28] Walsh B, Lynch M (2018). Evolution and selection of quantitative traits.

[29] Meuwissen THE, Sonesson AK, Gebregiwergis G, Woolliams JA (2020). Management of genetic diversity in the era of genomics. Front Genet.

[30] Neyhart JL, Lorenz AJ, Smith KP (2019). Multi-trait improvement by predicting genetic correlations in breeding crosses. G3 (Bethesda)..

[31] Hozé C, Baur A, Fritz S, Boichard D, editors. Prediction of gametic variance and its use in bovine breeding programs. In: Proceedings of the12th World Congress on Genetics Applied to Livestock Production: 3–8 July 2022; Rotterdam. 2022.

[32] Hickey JM, Chiurugwi T, Mackay I, Powell W, Hickey JM, Implementing Genomic Selection in CGIAR Breeding Programs Workshop Participants (2017). Genomic prediction unifies animal and plant breeding programs to form platforms for biological discovery. Nat Genet.

[33] Meuwissen THE (1997). Maximizing the response of selection with a predefined rate of inbreeding. J Anim Sci.

[34] Gorjanc G, Gaynor RC, Hickey JM (2018). Optimal cross selection for long-term genetic gain in two-part programs with rapid recurrent genomic selection. Theor Appl Genet.

[35] Gorjanc G, Hickey JM (2018). Alphamate: a program for optimizing selection, maintenance of diversity and mate allocation in breeding programs. Bioinformatics.

[36] Akdemir D, Sanchez JI (2016). Efficient breeding by genomic mating. Front Genet.

[37] Kinghorn BP (2011). An algorithm for efficient constrained mate selection. Genet Sel Evol.

[38] Yoshida GM, Yáñez JM, de Queiroz SA, Carvalheiro R (2020). Mate selection provides similar genetic progress and average inbreeding than optimum contribution selection in the long-term. Aquaculture.

[39] Zhang Z, Wang L (2022). A look-ahead approach to maximizing present value of genetic gains in genomic selection. G3 (Bethesda)..

[40] Moeinizade S, Wellner M, Hu G, Wang L (2020). Complementarity-based selection strategy for genomic selection. Crop Sci.

[41] Moeinizade S, Hu G, Wang L, Schnable PS (2019). Optimizing selection and mating in genomic selection with a look-ahead approach: an operations research framework. G3 (Bethesda)..

