# Supplementary figures and images for: Improving selection decisions with mating information by accounting for Mendelian sampling variances looking two generations ahead
Source: Genet Sel Evol. 2024 May 21;56:41. doi: 10.1186/s12711-024-00899-2 (PMC11107025; doi:10.1186/s12711-024-00899-2)

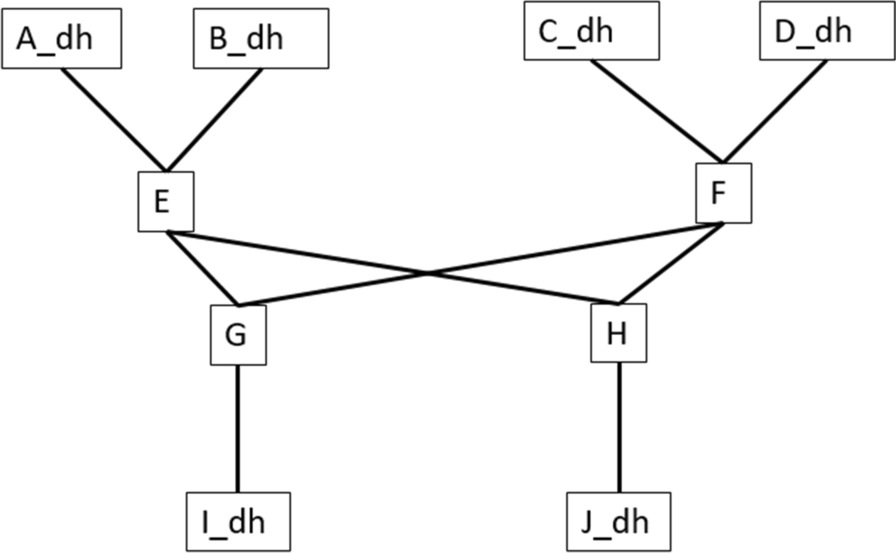

Supplement: Supplementary file 1 — Pedigree as considered in Fig. 4. The subscript “_dh” indicates that the individual is a double haploid. Individuals ”A_dh”, ”B_dh”, ”C_dh” and ”D_dh” are unrelated founders [file 12711_2024_899_Fig9_HTML.png]
