# Supplementary material for: Improving selection decisions with mating information by accounting for Mendelian sampling variances looking two generations ahead
Source: Genet Sel Evol. 2024 May 21;56:41. doi: 10.1186/s12711-024-00899-2 (PMC11107025; doi:10.1186/s12711-024-00899-2)
Supplement: Supplementary file 2 — Additional file 1: Text S1. Detailed explanation for the derivation of the ExpBVSelGrOff criterion. [file 12711_2024_899_MOESM1_ESM.docx]

Additional file 1 Text S1

Detailed explanation for the derivation of the ExpBVSelGrOff criterion

The following explains the calculation of the ExpBVSelGrOff criterion. ExpBVSelGrOff aims at describing the expected breeding value in selected grand-offspring.

$$ExpBVSelGrOff$$

$$=\frac{\frac{{BV}_{sire}+{BV}_{dam}}{2}+i_{O}*\sigma_{FS}+\bar{BV}+i_{p_{t}}*\sigma_{{BV}_{t}}+i_{p_{t+1}}*\sigma_{{BV}_{t+1}}}{2}$$

$$+i_{GO}*\sqrt{\frac{\left( 1-k_{O} \right)*\sigma_{FS}^{2}}{4}+\frac{\left( 1-k_{p_{t+1}} \right)*\sigma_{{BV}_{t+1}}^{2}}{4}+\bar{\sigma}_{{gamMS}_{off}}^{2}+\bar{\sigma}_{{gamMS}_{ind t+1}}^{2}}$$

The part highlighted in yellow describes the average breeding value of selected offspring of a mating ($\bar{BV}_{sel off}$) with $i_{O}$ as the selection intensity that is to be applied to the offspring.

The part highlighted in bright green describes the average breeding value of selected individuals of the population in the current generation ($\bar{BV}_{sel ind}$) with $i_{p_{t}}$ as the selection intensity that is to be applied to the individuals of the population in the current generation $t$ and $\sigma_{{BV}_{t}}$ as the standard deviation of breeding values in the current generation. The sum of the bright green and turquoise term describe the average breeding value of selected individuals of the population in the next generation (same generation as the offspring of the particular mating) with $i_{p_{t+1}}$ as the selection intensity to apply in the next generation and $\sigma_{{BV}_{t+1}}$ as the standard deviation of breeding values in the next generation.

The part highlighted in pink describes the variance of parent average breeding values for the grand-offspring of the mating in question. The sum of the pink part and the red part describes the variance of breeding values of grand-offspring ($\sigma_{GO}^{2}$) produced by selected offspring and selected members of the population of the offspring generation. $\bar{\sigma}_{{gamMS}_{ind t+1}}^{2}$ is the average gametic MSV of an individual in the population in the offspring generation. Since calculating this property accurately is impossible, we used the average gametic MSV of all individuals in the current generation as a proxy. Multiplying the selection intensity that is to be applied to grand-offspring with the standard deviation of breeding values of grand-offspring gives the selection differential that can be realized from the offspring to the grand-offspring generation.

In the notation above, we use different selection intensities depending on the type of animals and the generation in question. The above equation also distinguishes between the genetic variance of the population in the current generation and the offspring generation. When assuming selection intensities and genetic variances of the population to be constant over generations, the equation simplifies to the following.

$$ExpBVSelGrOff$$

$$=\frac{\frac{{BV}_{sire}+{BV}_{dam}}{2}+i_{O}*\sigma_{FS}+\bar{BV}+2i_{p}\sigma_{BV}}{2}$$

$$+i_{GO}*\sqrt{\frac{\left( 1-k_{O} \right)*\sigma_{FS}^{2}}{4}+\frac{\left( 1-k_{p} \right)*\sigma_{{BV}_{t+1}}^{2}}{4}+\bar{\sigma}_{{gamMS}_{off}}^{2}+\bar{\sigma}_{{gamMS}_{ind t+1}}^{2}}$$

$$=\frac{\frac{{BV}_{sire}+{BV}_{dam}}{2}+i_{O}*\sigma_{FS}+\bar{BV}+2i_{p}\sigma_{BV}}{2}+i_{GO}*\sigma_{GO}$$

When assuming the same selection intensity for the fullsib family, for the grand-offspring as well as for the population as we did in this study, the equation can be simplified further to the following:

$$ExpBVSelGrOff$$

$$=\frac{\frac{{BV}_{sire}+{BV}_{dam}}{2}+i_{O}*\sigma_{FS}+\bar{BV}+2i_{p}\sigma_{BV}}{2}+i_{GO}*\sigma_{GO}$$

$$=\frac{\frac{{BV}_{sire}+{BV}_{dam}}{2}+i_{O}*\sigma_{FS}+\bar{BV}}{2}+i_{p}\sigma_{BV}+i_{GO}*\sigma_{GO}$$

$$=\frac{{BV}_{sire}+{BV}_{dam}}{4}+\frac{\bar{BV}_{t+0}}{2}+i_{p}*\left( 0.5*\sigma_{FS}+\sigma_{BV}+\sigma_{GO} \right)$$

The equation as above describes the expected breeding value of selected grand-offspring. Since the average breeding value of the population is the same for all evaluated matings, it does not add anything in distinguishing matings and can thus be omitted. This may become more intuitive when thinking about the population average breeding value as being 0. In addition, the standard deviation of breeding values in the population ($\sigma_{BV}$) is identical for all evaluated matings and can thus also be omitted. Consequently, we arrive at the final notation with:

$$ExpBVSelGrOff=0.25*\left( {BV}_{sire}+{BV}_{dam} \right)+i_{p}*\left( 0.5*\sigma_{FS}+\sigma_{GO} \right)$$
