# Supplementary material for: Improving selection decisions with mating information by accounting for Mendelian sampling variances looking two generations ahead
Source: Genet Sel Evol. 2024 May 21;56:41. doi: 10.1186/s12711-024-00899-2 (PMC11107025; doi:10.1186/s12711-024-00899-2)
Supplement: Supplementary file 3 — Additional file 2: Figure S1. Differences in the decisions made for selection based on the ProbSelOff and ExpBVSelOff criteria. [file 12711_2024_899_MOESM2_ESM.docx]

Additional file 2 Figure S1

Differences in the decisions made for selection based on the ProbSelOff and ExpBVSelOff criteria

| 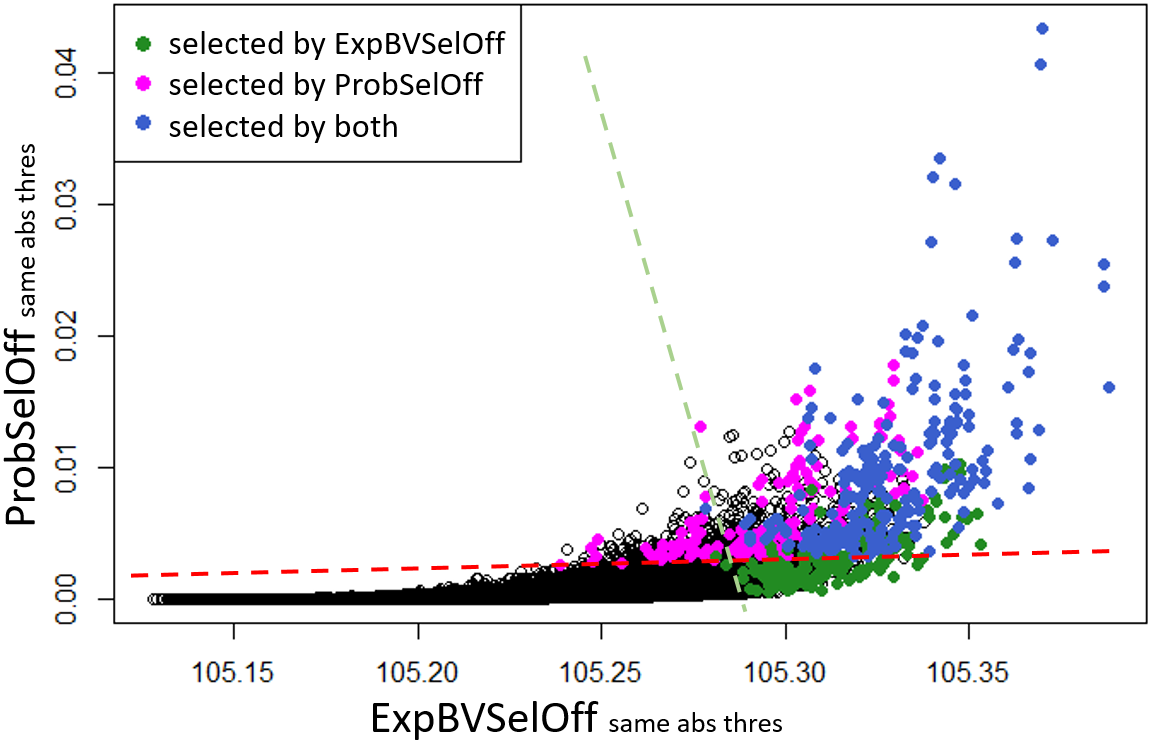 |
| --- |
| Additional file 2 Figure S1: Comparison of the decisions made for selection based on ExpBVSelOff and ProbSelOff in a random replicate when the absolute truncation selection point is the same for all matings. Every point corresponds to a mating that is possible between a particular pair of selection candidates. Green and blue points correspond to matings that are possible between animals selected based on the ExpBVSelOff criterion. Pink and blue points indicate matings that are possible between animals selected by the ProbSelOff criterion. Thus, the blue points show the overlap between the decisions. The dashed red and green lines indicate imaginary cutoff lines for each of the criteria to aid visualization. |
